# Supplementary material for: Effective in vivo binding energy landscape illustrates kinetic stability of RBPJ-DNA binding
Source: Nat Commun. 2025 Feb 1;16:1259. doi: 10.1038/s41467-025-56515-4 (PMC11787368; doi:10.1038/s41467-025-56515-4)
Supplement: Supplementary file 1 — Supplementary Information [file 41467_2025_56515_MOESM1_ESM.pdf]

Supplementary Information

Effective *in vivo* binding energy landscape illustrates kinetic stability of RBPJ-DNA binding

Duyen Huynh[1,*], Philipp Hoffmeister[2,*], Tobias Friedrich[3,4,5], Kefan Zhang[1], Marek Bartkuhn[4,5], Francesca Ferrante[3], Benedetto Daniele Giaimo[3], Rhett A. Kovall[6], Tilman Borggrefe[3], Franz Oswald[2,#], J. Christof M. Gebhardt[1,#]
(* equal contribution, # corresponding author)

[1]Institute of Experimental Physics and IQST, Ulm University, Albert-Einstein-Allee 11, 89081 Ulm, Germany
[2]Clinic of Internal Medicine I, University Medical Center Ulm, Albert-Einstein-Allee 23, 89081 Ulm, Germany
[3]Institute of Biochemistry, Justus-Liebig-Universität Gießen, Friedrichstraße 24, 35392 Gießen, Germany
[4]Biomedical Informatics and Systems Medicine, Justus-Liebig-Universität Gießen, Aulweg 128, 35392 Gießen, Germany
[5]Institute for Lung Health (ILH), Aulweg 132, 35392 Gießen, Germany
[6]Department of Molecular Genetics, Biochemistry and Microbiology, University of Cincinnati College of Medicine, Cincinnati, OH, USA
# to whom correspondence should be addressed: franz.oswald@uni-ulm.de, christof.gebhardt@uni-ulm.de

Contents

SUPPLEMENTARY FIGURES 2
SUPPLEMENTARY TABLES 23

Supplementary Figures

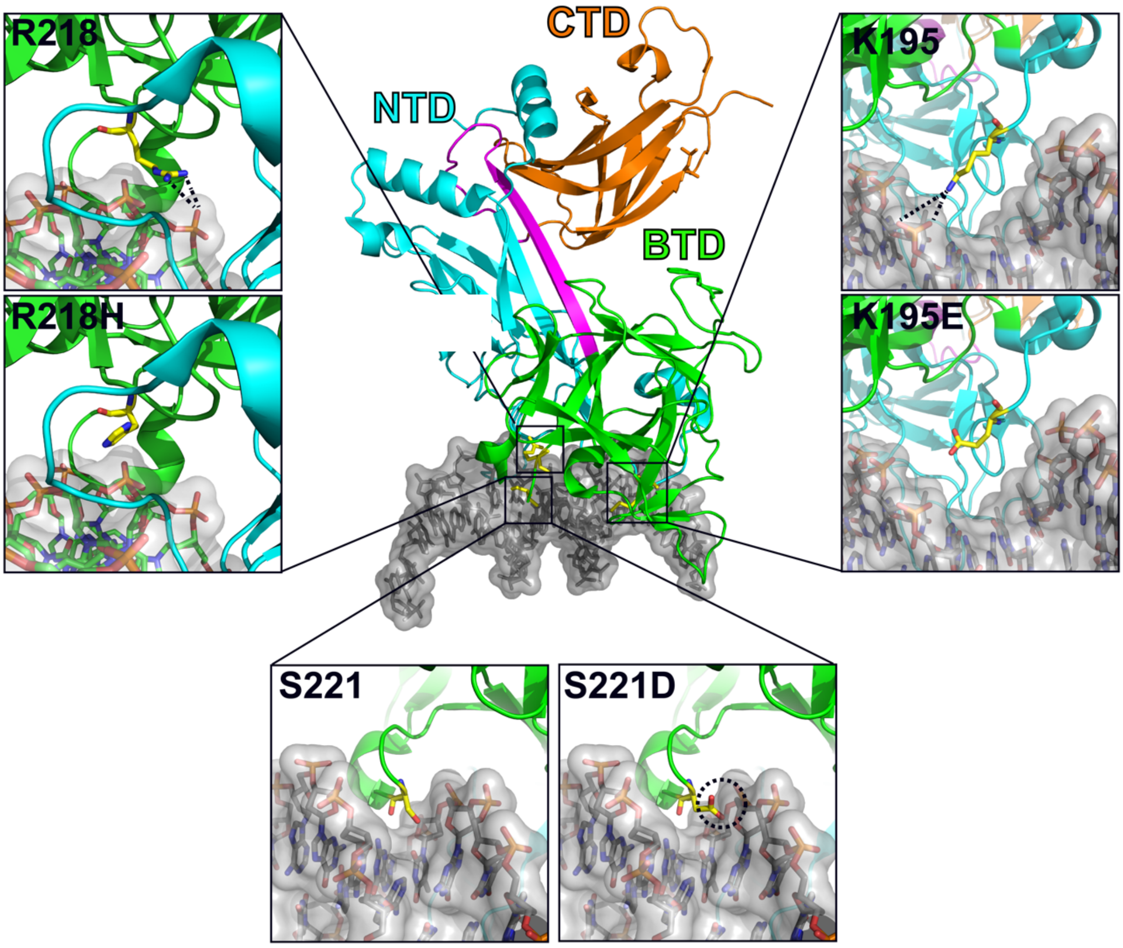

Supplementary Figure 1: Spatial orientation of DNA binding interface mutations of RBPJ

Structure of mouse RBPJ in complex with the HES1 non-consensus site (PDB entry: 3IAG) with highlighted NTD (cyan), BTD (green) and CTD (orange) as well as the DNA (grey). Zooms: Spatial orientation of the amino acids K195, R218 and S221 and of the DNA-binding deficient mutations K195E, R218H and S221D. Structures and mutations of single amino acids were performed using PyMOL.

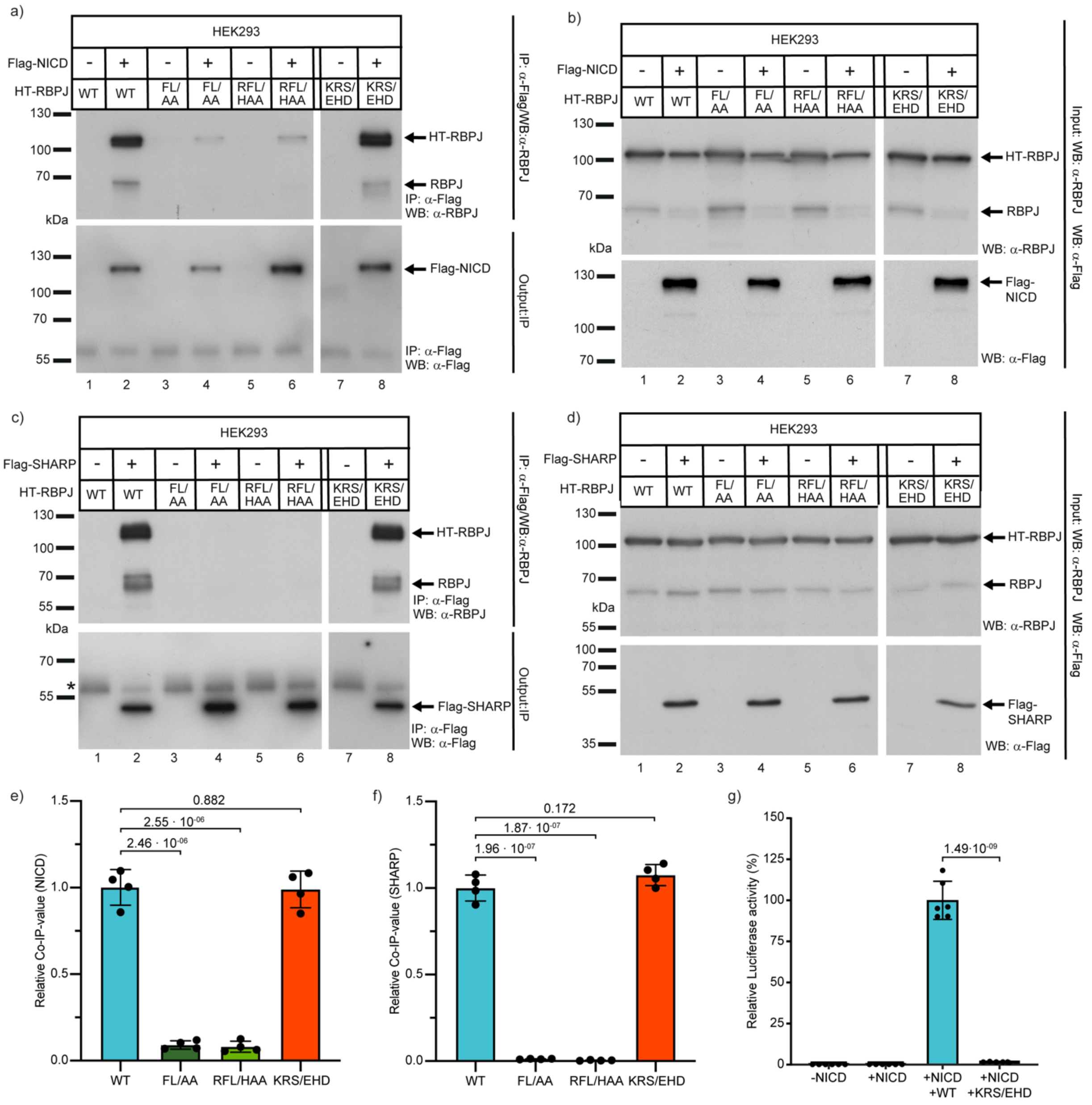

Supplementary Figure 2: Binding of HT-RBPJ variants to NICD and SHARP

a) Upper panel: Western blot of Co-IP between Flag-NICD and HT-RBPJ variants. HEK293 cells were co-transfected with expression plasmids for Flag-tagged NICD1 and Halo-tagged RBPJ-WT, FL/AA, RFL/HAA or KRS/EHD. Co-IP was performed 24 h after transfection using an anti-Flag antibody. HT-RBPJ-WT as well as HT-RBPJ-KRS/EHD show similar interaction with Flag-NICD1. HT-RBPJ mutants FL/AA and RFL/HAA deficient in cofactor binding show a strongly reduced interaction with Flag-NICD1. Lower panel: Western blot showing the precipitated Flag-NICD protein (output). * marks the band of the heavy chain of the anti-Flag antibody used for precipitation. For unprocessed Western blots see Source Data file. For abbreviation of mutations see main text.

b) Western blot to verify the expression of Halo-tagged RBPJ variants (upper panel) and Flag-tagged NICD1 (lower panel) in cells used for the Co-IP in a). For unprocessed Western blot see Source Data file. For abbreviation of mutations see main text.

c) Upper panel: Western blot of Co-IP between Flag-SHARP and HT-RBPJ variants. HEK293 cells were co-transfected with expression plasmids for Flag-tagged SHARP and

Halo-tagged RBPJ-WT, FL/AA, RFL/HAA, and KRS/EHD. Co-IP was performed 24 h after transfection using an anti-Flag antibody. HT-RBPJ-WT and HT-RBPJ-KRS/EHD show similar interaction with Flag-SHARP. HT-RBPJ mutants FL/AA and RFL/HAA deficient in cofactor binding show no interaction with Flag-SHARP. Lower panel: Western blot showing the precipitated Flag-NICD protein (output). * marks the band of the heavy chain of the anti-Flag antibody used for precipitation. For unprocessed Western Blot see Source Data file. For abbreviation of mutations see main text.

d) Western blot to verify the expression of Halo-tagged RBPJ variants (upper panel) and Flag-tagged SHARP (lower panel) in cells used for the Co-IP in d). For unprocessed Western blot see Source Data file. For abbreviation of mutations see main text.

e) Relative co-precipitation values for Co-IP with NICD calculated as described in the materials and methods section (mean values ± s.d.; n= 4 independent experiments).

f) Relative co-precipitation values for Co-IP with SHARP calculated as described in the materials and methods section (mean values ± s.d.; n= 4 independent experiments).

g) In vivo Luciferase activity assays. Relative luciferase activity of RBPJ-specific reporter constructs in RBPJ knockout cell line transfected with Halo-tagged RBPJ-WT or KRS/EHD and co-transfected with NICD (mean values ± s.d; n= 4.independent experiments). P-values were determined using two-tailed, unpaired Student's t-test. -/+NICD: control with untransfected / NICD-transfected RBPJ knockout cell line.

Data are provided as Source data file for Supplementary figure 2e, f, g.

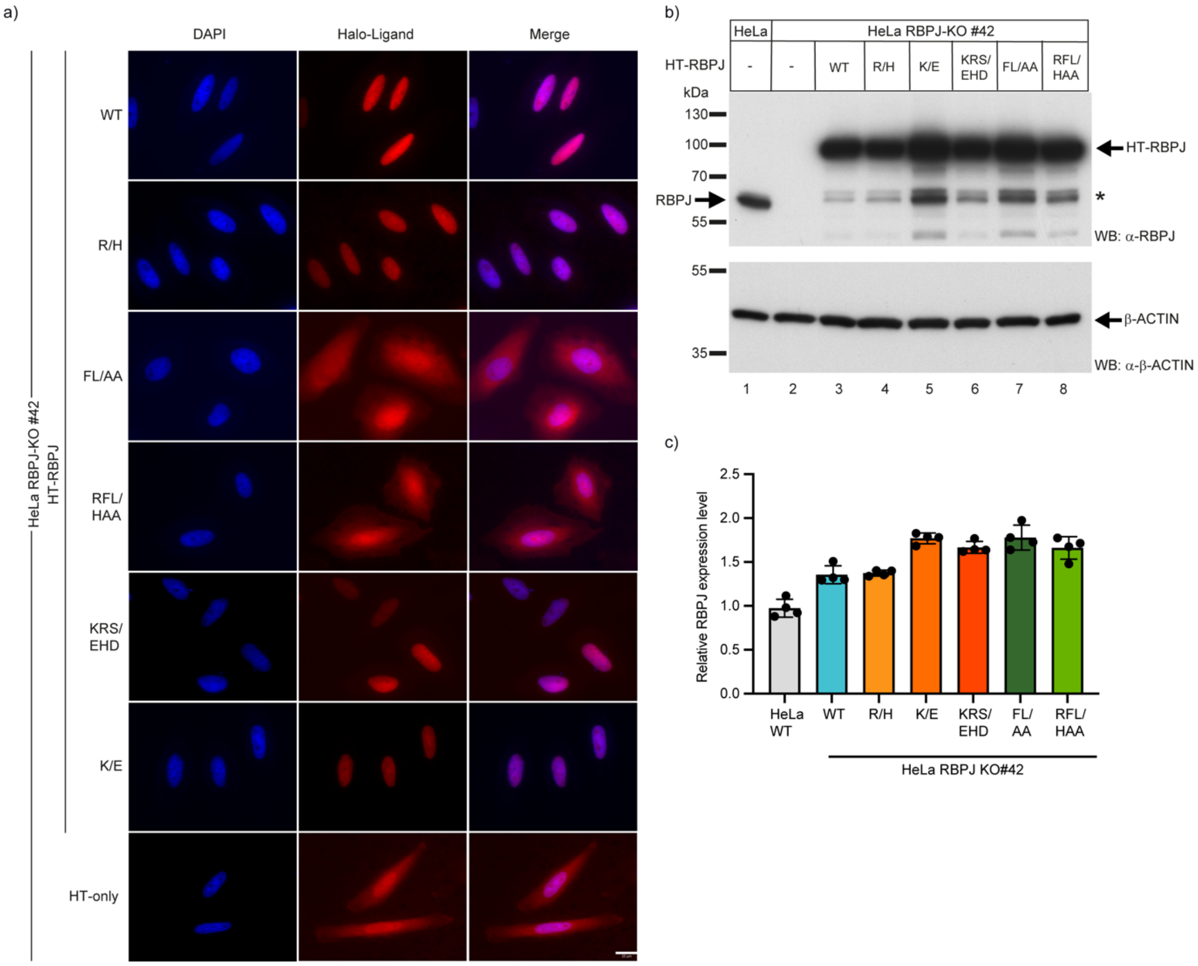

Supplementary Figure 3: Characterization of RBPJ-depleted HeLa cell lines with reintroduced RBPJ variants

a) Fluorescence images of RBPJ-depleted HeLa cells (clone #42) stably expressing HT-RBPJ-WT, HT-RBPJ variants or the HaloTag alone. Nuclei were labeled with DAPI (Column 1) and HaloTag was fluorescently labeled with HTL-TMR (Column 2). Merged images (Column 3) revealed nuclear distribution of HT-RBPJ-WT and RBPJ variants with mutations in the DNA binding interface only (R/H, KRS/EHD and K/E). RBPJ variants with mutations in the cofactor binding interface (FL/AA and RFL/HAA) as well as HaloTag alone showed both cytoplasmic and nuclear distribution. Images were taken with a fluorescence microscope with a 63x lens and the scale bar represents 20 μm. For abbreviation of mutations see main Text.

b) Verification of stable expression of HT-RBPJ-WT and variants in RBPJ-depleted HeLa cell line #42 using Western blot. Lanes 2-7 contained lysates from cell lines expressing the following RBPJ variants, respectively: WT, R/H, K/E, KRS/EHD, FL/AA and RFL/HAA. Lane 1 contained lysate of RBPJ-depleted cells and was used as negative control. A specific α-RBPJ antibody was used for blotting. Detection of β-ACTIN was used as loading control using a specific α-β-ACTIN antibody. Arrows indicate bands for HaloTag-RBPJ or β-ACTIN. For unprocessed Western Blot see Source Data file. For abbreviation of mutations see main text.

c) Quantification of relative expression levels of HT-RBPJ variants in HeLa RPBJ knock-out cells (clone #42) compared to endogenous RBPJ expression in HeLa WT cells (mean values ± s.d.; n= 4 independent experiments).

Data are provided as Source data file for Supplementary figure 3c.

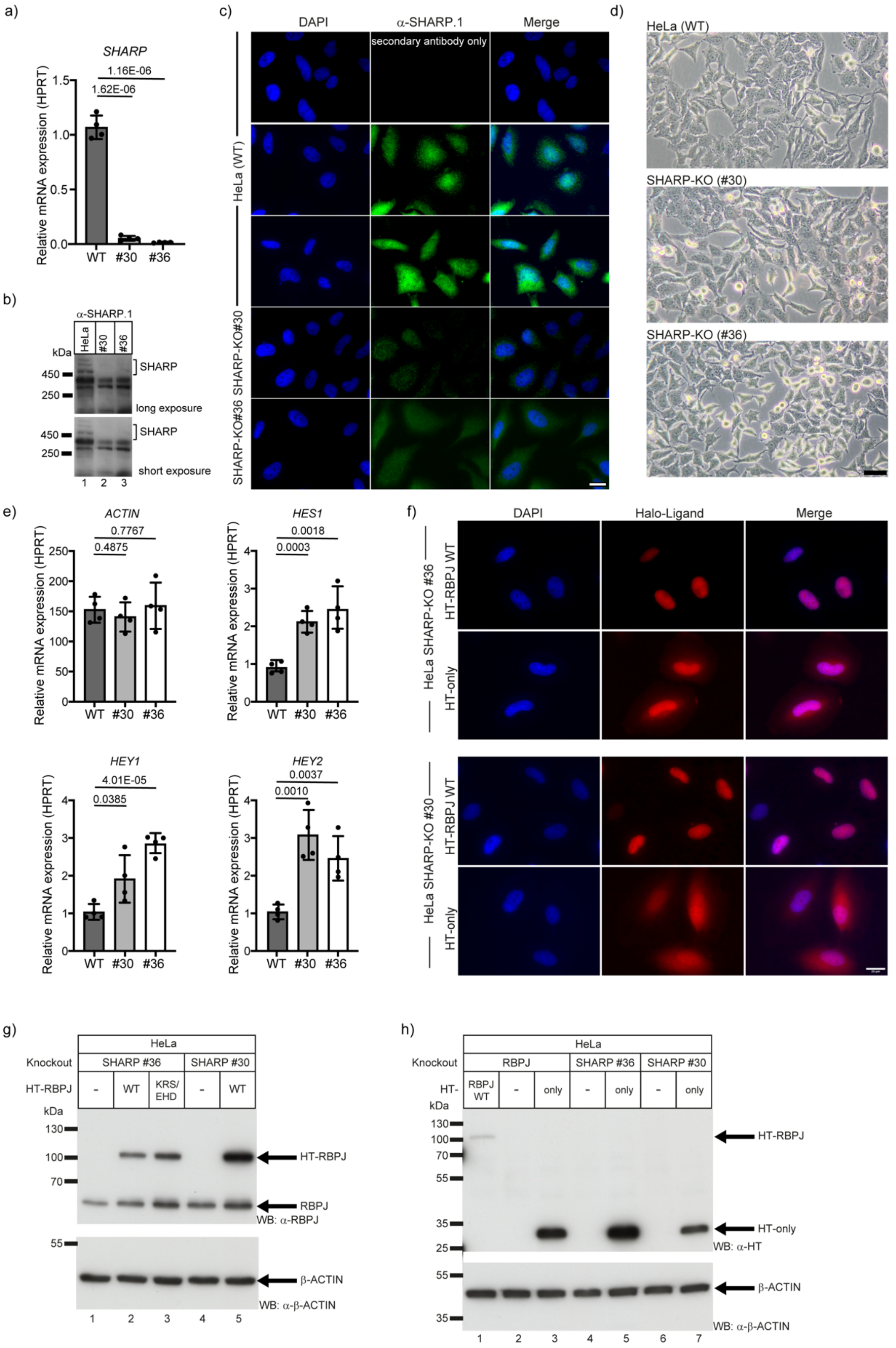
a)
SHARP
1.16E-06
1.62E-06
Relative mRNA expression (HPRT)
WT #30 #36
b)
α-SHARP.1
HeLa #30 #36
kDa
450
250
SHARP
long exposure
short exposure
1 2 3
c)
DAPI
α-SHARP.1
Merge
secondary antibody only
HeLa (WT)
SHARP-KO#36 SHARP-KO#30
d)
HeLa (WT)
SHARP-KO (#30)
SHARP-KO (#36)
e)
ACTIN
0.7767
0.4875
HES1
0.0018
0.0003
HEY1
4.01E-05
0.0385
HEY2
0.0037
0.0010
Relative mRNA expression (HPRT)
WT #30 #36
f)
DAPI
Halo-Ligand
Merge
HeLa SHARP-KO #36
HT-RBPJ WT
HT-only
HeLa SHARP-KO #30
g)
HeLa
Knockout
SHARP #36
SHARP #30
HT-RBPJ
-
WT
KRS/ EHD
kDa
130
100
70
55
HT-RBPJ
RBPJ
WB: α-RBPJ
β-ACTIN
WB: α-β-ACTIN
1 2 3 4 5
h)
HeLa
Knockout
RBPJ
SHARP #36
SHARP #30
HT-
RBPJ WT
only
kDa
130
100
70
55
35
25
HT-RBPJ
HT-only
WB: α-HT
β-ACTIN
WB: α-β-ACTIN
1 2 3 4 5 6 7

Supplementary Figure 4: Characterization of SHARP-depleted HeLa cell lines

a) Relative expression of SHARP mRNA in control and SHARP-depleted HeLa cell lines (clones #30 and #36). Expression was determined by qPCR and calculated relative to the housekeeping gene HPRT. SHARP-depleted cells display almost full depletion of SHARP expression. Shown is the mean value ± s.d., n= 4 independent experiments, each containing at least 1 technical replicate. P-values were determined using two-tailed, unpaired Student's t-test.

b) Detection of SHARP in control and SHARP-depleted HeLa cells by Western blotting. Two distinct bands were detected at the height of ~500 kDa in HeLa control cells by using an α-SHARP.1 antibody. Long- and short-term exposures are displayed in the upper and lower panel, respectively.

c) Immunofluorescence microscopy images using the α-SHARP.1 antibody. Images were taken with a 63x lens and the scale bar represents 20 µm. Control and SHARP-depleted HeLa cells #30 and #36 were stained with DAPI for visualization of the nucleus (Column 1). As negative control, HeLa control cells were incubated only with secondary antibodies (row 1), while the other samples were additionally incubated with a primary α-SHARP.1 antibody (row 2 to 5). SHARP-specific fluorescence signals are shown in column 2. Merged images (Column 3) show the subcellular localization of SHARP in the nucleus and in the cytoplasm in all cell lines, however, SHARP-depleted cells #30 and #36 show a reduced fluorescent signal (Row 4 and 5).

d) Bright-field microscopy images of control (top panel) and SHARP-depleted HeLa cells #30 (middle panel) and #36 (bottom panel). The images were taken using a 20x lens and the scale bar represents 50 µm.

e) mRNA expression levels of Notch target genes *HES1*, *HEY1* and *HEY2* as well as of the housekeeping gene *ACTIN* were determined by qPCR. HeLa control and HeLa SHARP-depleted cells #30 and #36 were examined. Except for the housekeeping gene *ACTIN*, SHARP-depleted cells exhibited increased mRNA levels of Notch target genes compared to HeLa control cells due to derepression. Shown are the means ± s.d. n= 4 independent experiments, each containing at least 1 technical replicate. P-values were determined using two-tailed, unpaired Student's t-test.

f) Fluorescence microscopy images of SHARP-depleted HeLa cell lines #36 and #30 stably expressing HT-RBPJ-WT or just the HaloTag. The nuclei were stained with DAPI (Column 1), HaloTag was labeled with HTL-TMR (Column 2). Merged images (Column 3) show that HT-RBPJ-WT is located exclusively in the nucleus, HaloTag alone is located in both the cytoplasm and the nucleus.

g) Verification of stable expression of HT-RBPJ-WT and HT-RBPJ-KRS/EHD using Western blotting. Lanes 2 and 3 contained lysates from SHARP-depleted HeLa cells #36 expressing either HT-RBPJ-WT or HT-RBPJ-KRS/EHD. Lane 5 contained lysate from SHARP-depleted HeLa cells #30 expressing HT-RBPK-WT. Lane 1 and 4 contained lysates of SHARP-depleted cells #36 and #30 as negative controls. A specific α-RBPJ antibody was used. Detection of β-ACTIN was used as loading control using a specific α-β-ACTIN antibody. Arrows indicate bands for endogenous RBPJ, HT-RBPJ-WT or β-ACTIN. Unprocessed Western Blots are provided as Source Data file.

h) Verification of stable expression of HT-RBPJ-WT or HaloTag protein using Western blotting. Lanes 3, 5 and 7 contained lysates from RBPJ-depleted, SHARP-depleted #36 or SHARP-depleted #30 cell lines expressing the HaloTag protein. Lanes 2, 4 and 6 contained lysates from RBPJ-depleted, SHARP-depleted #36 or SHARP-depleted #30 cell lines respectively as negative controls. Lane 1 contained lysate from a RBPJ-depleted cell line expressing HT-RBPJ-WT as a positive control. A specific α-HaloTag antibody was used. Detection of β-ACTIN was used as loading control using a specific α-β-ACTIN antibody. Arrows indicate bands for HaloTag protein, HT-RBPJ-WT or β-ACTIN. For unprocessed Western Blot see Source Data file.

Source data are provided as Source Data file for Supplementary Figure 4a, e.

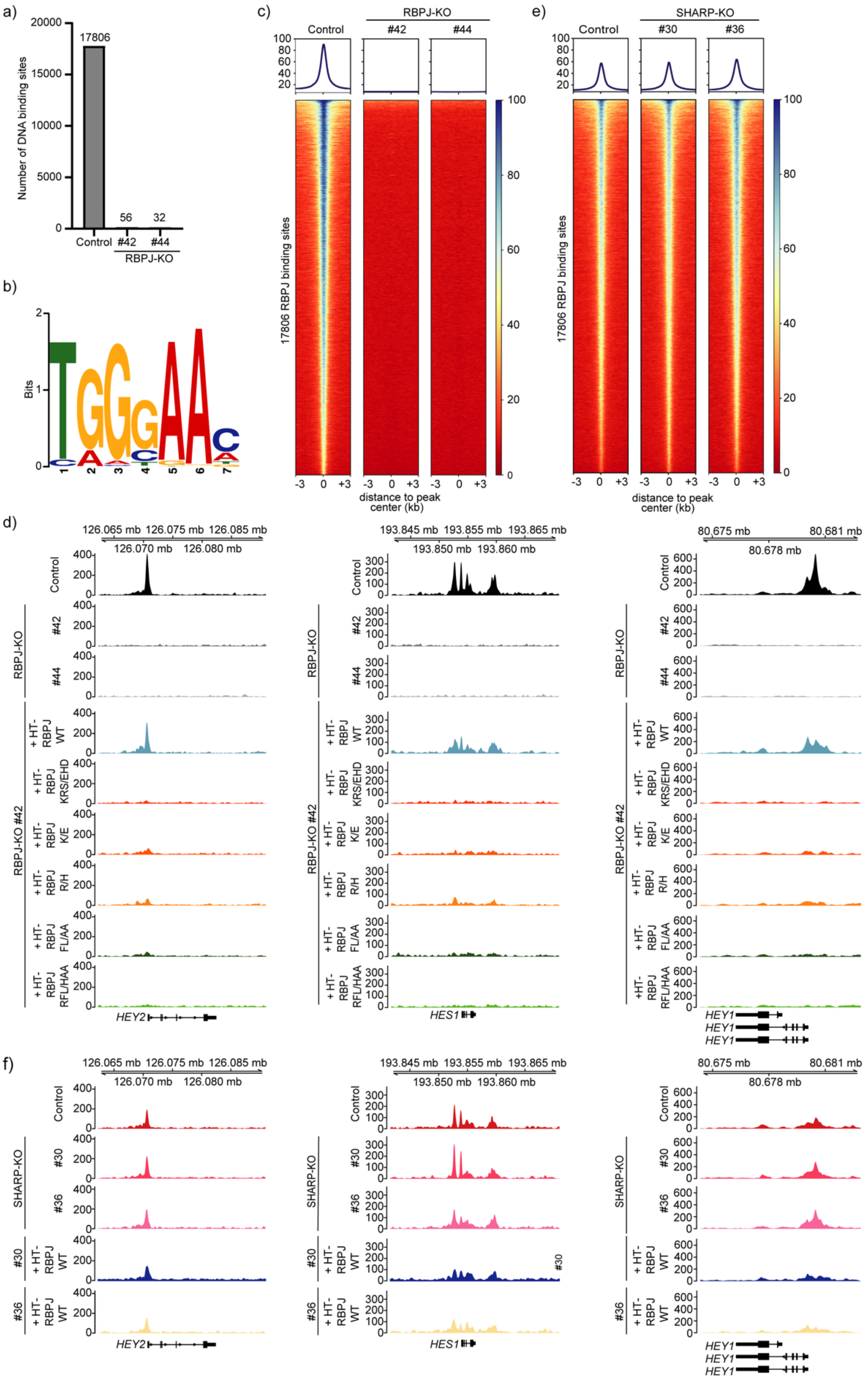

Supplementary Figure 5: Expression profiles of RBPJ-depleted and SHARP-depleted cell lines

a) Number of DNA binding sites identified in RBPJ ChIP-Seq for control HeLa cells transfected with an empty vector and RBPJ-depleted HeLa cell lines clones #42 and #44. In

contrast to control HeLa cells, the RBPJ-depleted cell lines showed absence of RBPJ binding events.

b) DNA-binding sequence logo obtained from 17806 RBPJ binding sites identified in HeLa control cells. The RBPJ consensus sequence is significantly enriched.

c) Heatmap of RBPJ ChIP-Seq reads covering 3 kb up- and downstream of binding sites for all 17806 binding sites identified in control HeLa cells transfected with an empty vector. In contrast to the control HeLa cells (Left column), the RBPJ-depleted cell lines clone #42 (Middle column) and clone #44 (Right column) showed absence of RBPJ binding events.

d) Genome browser sections of the location indicated on top showing the ChIP-Seq coverage for promoter or enhancer regions of the Notch target genes *HEY2* (left panel), *HES1* (middle panel), and *HEY1* (right panel) for RBPJ-depleted cells clones #42 and #44 and for RBPJ-depleted cells stably expressing the indicated HT-RBPJ variants.

e) Heatmap of RBPJ ChIP-Seq reads covering 3 kb up- and downstream of binding sites for all 17806 binding sites identified in control HeLa cells transfected with an empty vector. Compared to the control cell line (Left column), the two SHARP-depleted cell lines clones #30 (Middle column) and #36 (Right column) showed a similar profile of RBPJ binding events.

f) Genome browser sections of the location indicated on top showing the ChIP-Seq coverage for promoter or enhancer regions of the Notch target genes *HEY2* (left panel), *HES1* (middle panel), and *HEY1* (right panel) for the SHARP-depleted cells clones #30 and #36 and for SHARP-depleted cells stably expressing HT-RBPJ-WT.

a)

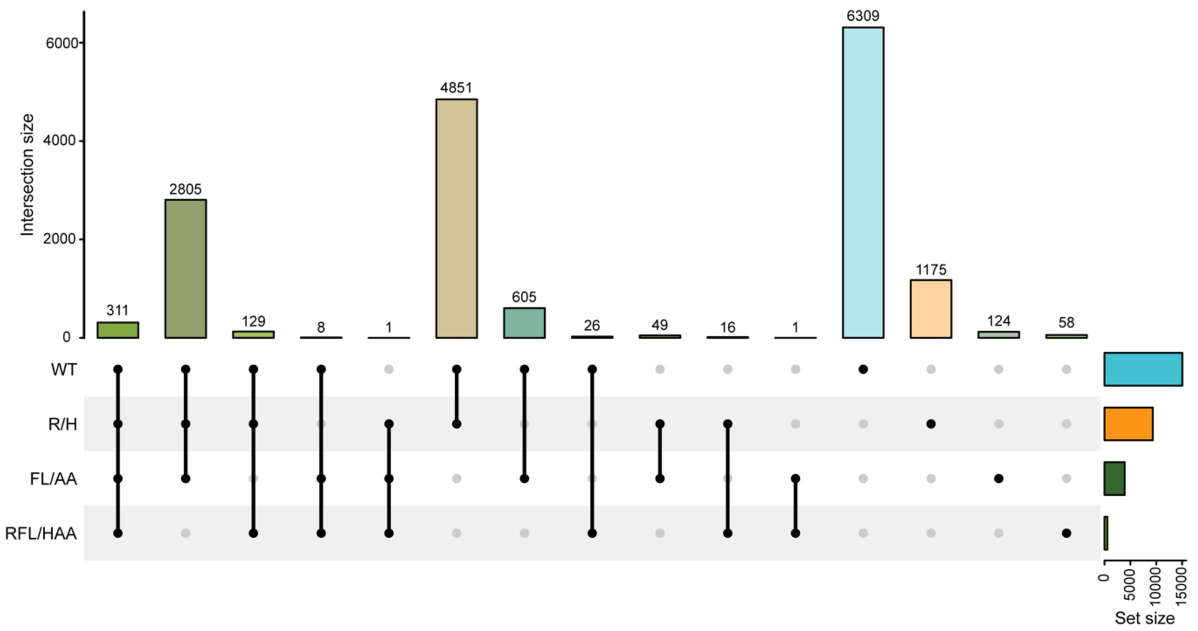

b)

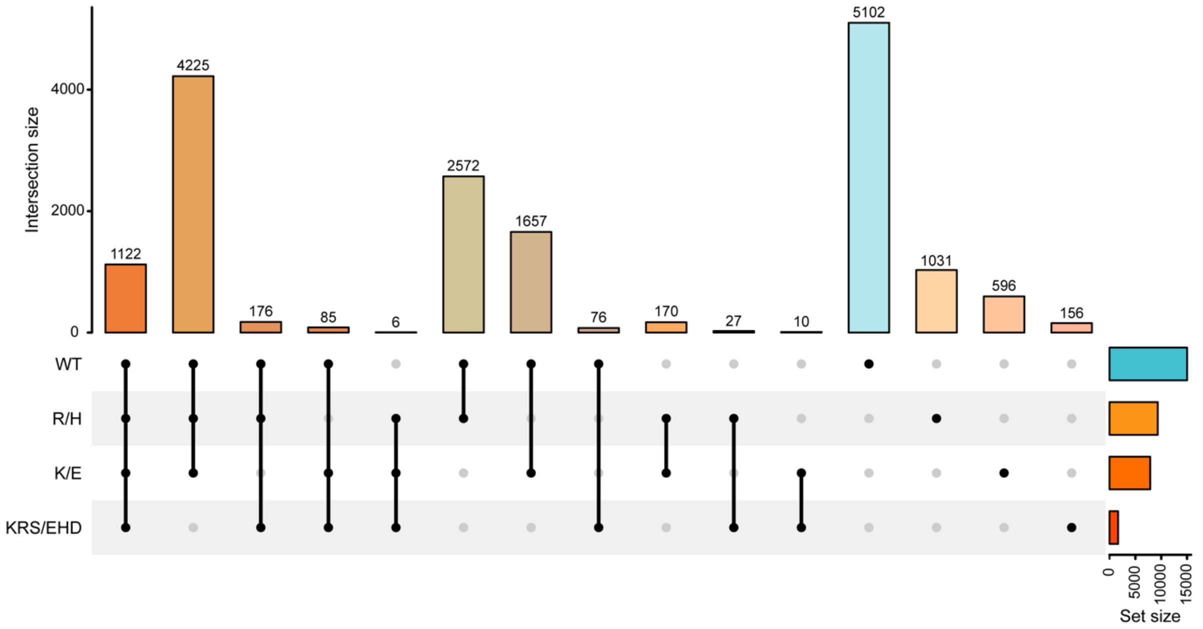

Supplementary Figure 6: Common binding sites among HT-RBPJ variants

a) UpSet plot representing common binding sites among HT-RBPJ-WT and the HT-RBPJ mutants R/H, FL/AA, and RFL/HAA in a HeLa RBPJ knockout cellline (clone #42). The plot corresponds to the Venn diagram in Figure 1 f). Set size corresponds to binding sites in Figure 1 e).

b) UpSet plot representing common binding sites among HT-RBPJ-WT and the HT-RBPJ DNA binding mutants R/H, K/E, and KRS/EHD in a HeLa RBPJ knockout cellline (clone #42). The plot corresponds to the Venn diagram in Figure 1 g). Set size corresponds to binding sites in Figure 1 e).

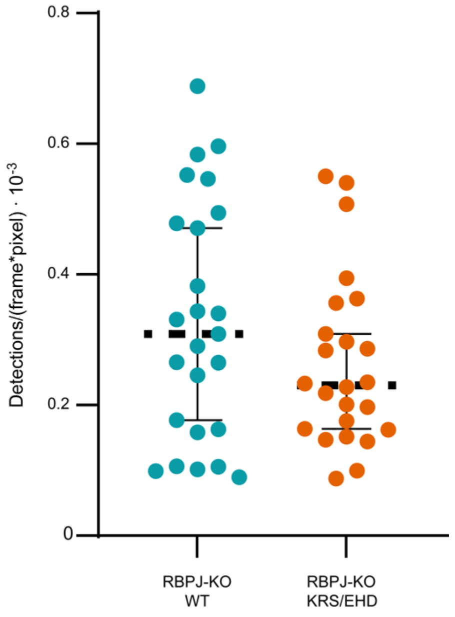

Supplementary Figure 7: Number of detected molecules

Average number of detected single molecules per frame of 11.7 ms continuous movies for HT-RBPJ-WT and KRS/EHD in RBPJ knockout cells. Dashed black lines indicate the median value and the solid lines the 0.25 and 0.75 quartiles. Experimental statistics are listed in Supplementary Table 5.

Data are provided as Source Data file for Supplementary Figure 7.

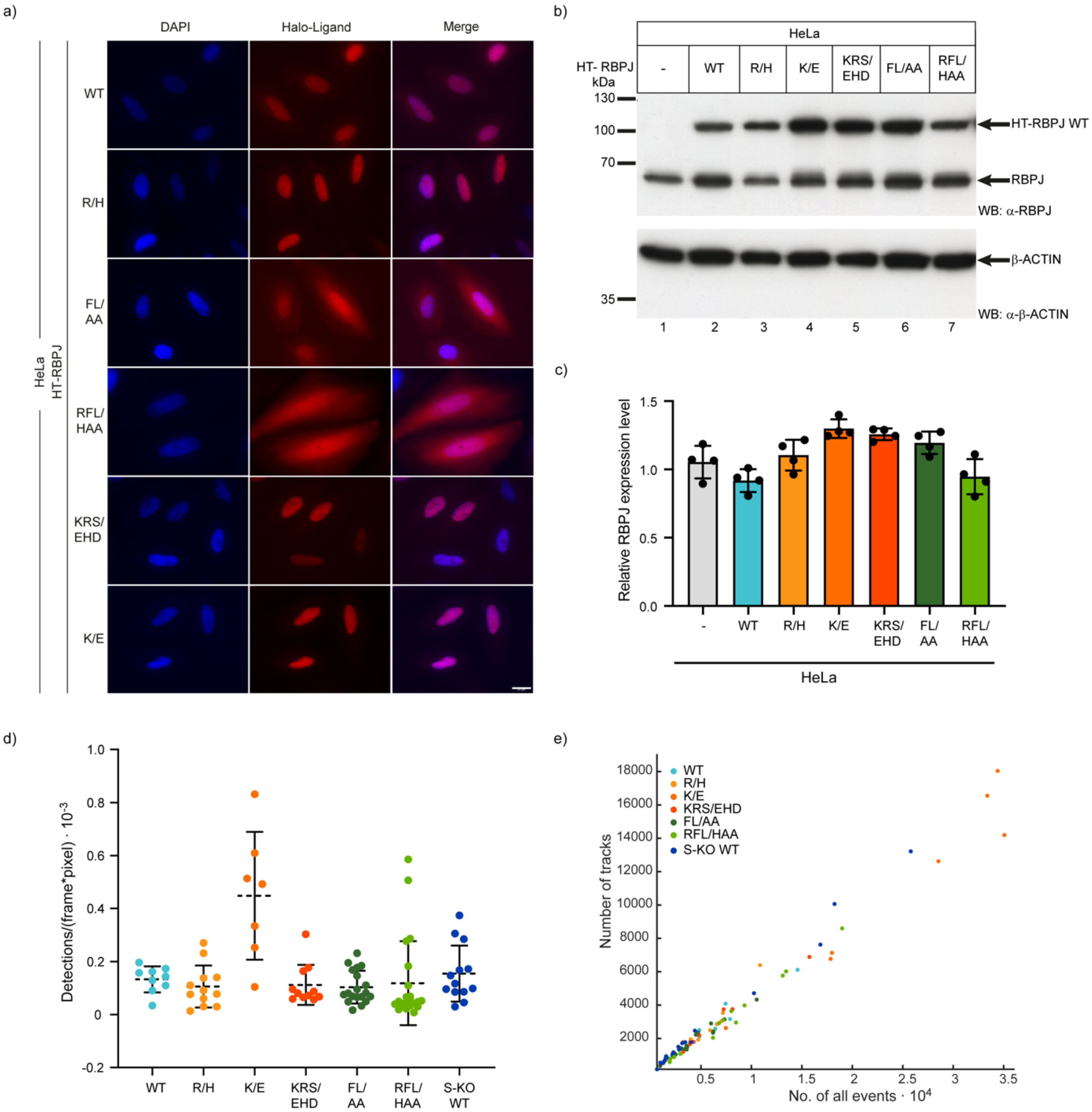

Supplementary Figure 8: Quantification of expression level of HT-RBPJ variants

a) Fluorescence images of HeLa cells stably expressing HT-RBPJ variants. The nuclei were labeled with DAPI (Column 1) and HaloTag was fluorescently labeled with HTL-TMR (Column 2). Merged images (Column 3) revealed that HT-RBPJ-WT and mutants of the DNA binding interphase (R/H, KRS/EHD, and K/E) were localized exclusively in the nucleus (row 1, 2, 5 and 6). HT-RBPJ mutants FL/AA and RFL/HAA were localized in both the cytoplasm and the nucleus (row 3 and 4). Images were taken with a fluorescence microscope with a 63x lens and the scale bar represents 20 μm.

b) Verification of stable expression of HT-RBPJ variants using Western blot. Lanes 2-7 contained lysates from HeLa cell lines expressing the following HT-RBPJ proteins respectively: WT, R/H, K/E, KRS/EHD, FL/AA and RFL/HAA. Lane 1 contained lysate of untreated HeLa cells as negative control. A specific α-RBPJ antibody was used. Detection of β-Actin was used as loading control using a specific α-β-Actin antibody. Arrows indicate bands of endogenous RBPJ, HT-RBPJ or β-Actin. For unprocessed Western blot see Source Data. For abbreviation of mutations see main text.

c) Quantification of relative expression levels of HT-RBPJ variants in HeLa cells compared to endogenous RBPJ expression (mean values ± s.d.; n= 4 independent experiments).

d) Average number of detected single-molecule spots per frame for indicated HT-RBPJ variants. Dashed black lines indicate the median value and the solid lines the 0.25 and 0.75 quantiles. Experimental statistics are listed in Supplementary Table 5.

e) Number of identified single-molecule tracks versus the number of detected spots per frame for indicated HT-RBPJ variants. Data shown are from 10 ms continuous movies. Experimental statistics are listed in Supplementary Table 5.

Source data are provided as Source Data file for Supplementary Figure 8c, d and e.

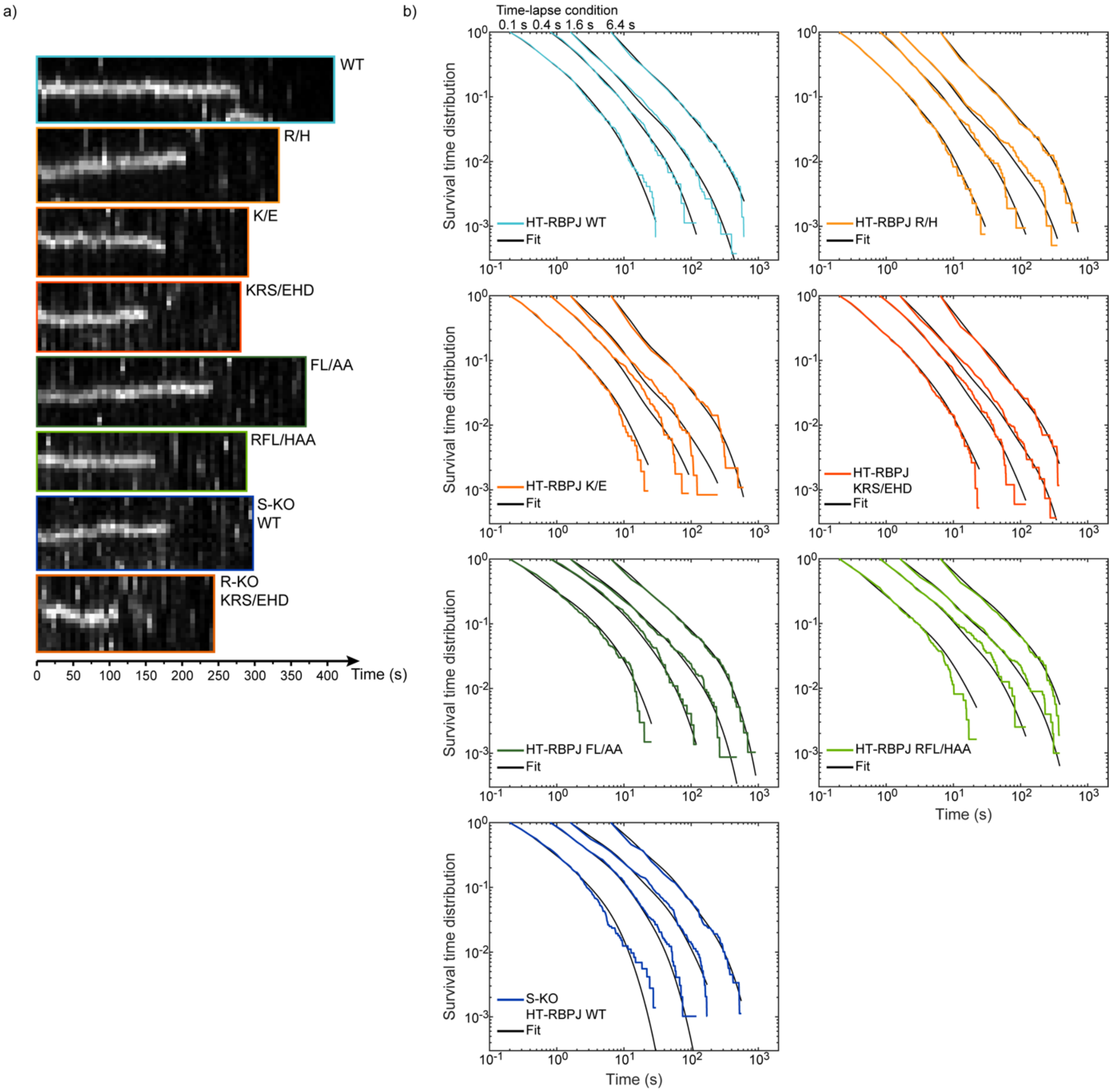

Supplementary Figure 9: Survival time distributions of HT-RBPJ variants

a) Example kymographs of a bound HT-RBPJ molecule of the indicated variant from 6.4 s time-lapse measurements.

b) Survival time distributions HT-RBPJ variants at time-lapse conditions shown on top and survival time function obtained with GRID (black lines). Experimental statistics are listed in Supplementary Table 3.

Source data are provided as Source Data file for Supplementary Figure 9b.

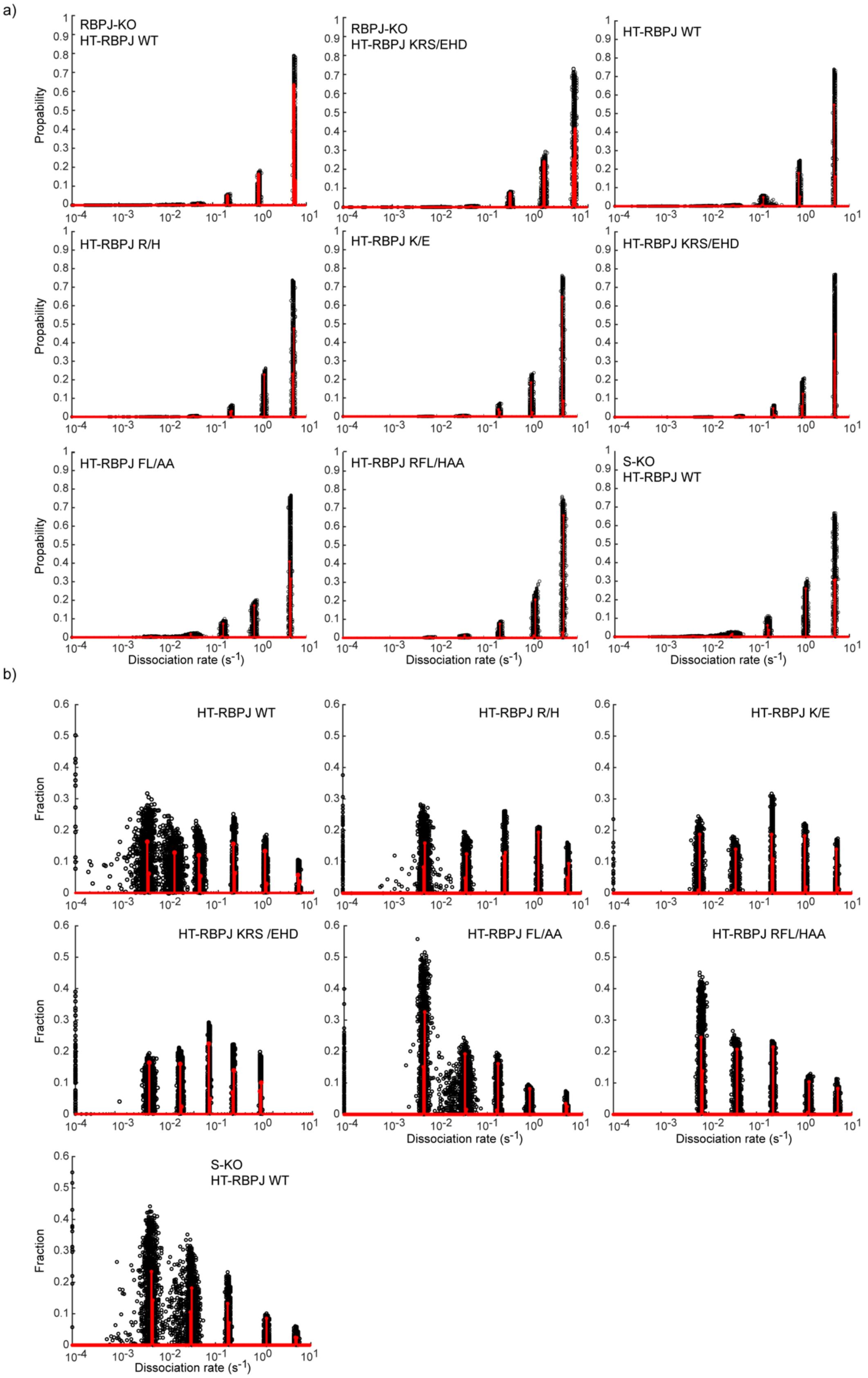

Supplementary Figure 10: GRID dissociation rate spectra of HT-RBPJ variants

a) Event and **b)** state spectrum of HT-RBPJ variants obtained with GRID. Resampling was performed by repeating GRID analysis for 499 times using 80% of data for each run.

Experimental statistics are listed in Supplementary Table 3. Source data are provided as Source Data file for Supplementary Figure 10a, b.

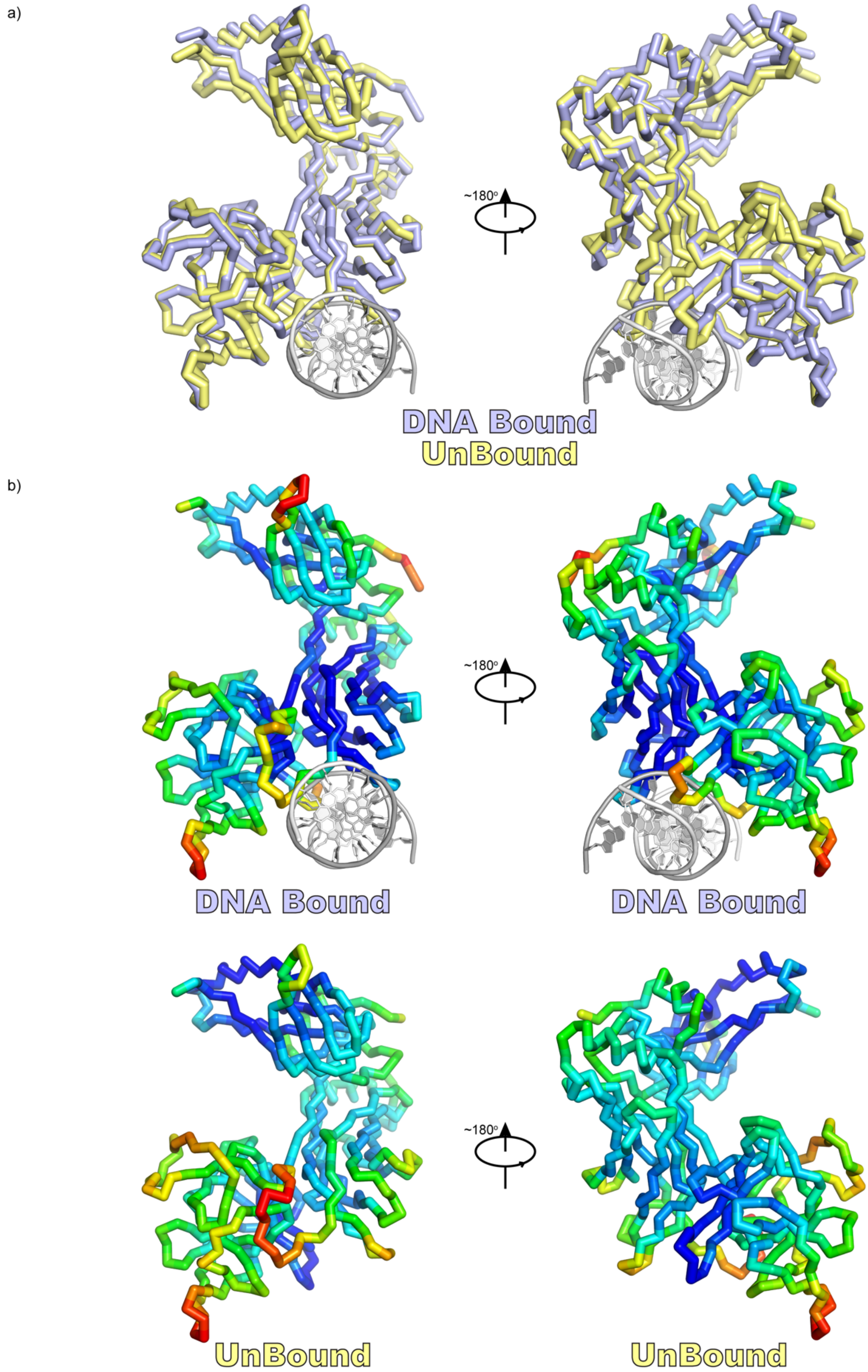

Supplementary Figure 11: Structural Comparison of RBPJ [Su(H)] in DNA-bound and -unbound states

The figure shows a structural overlay and comparison of the two RBPJ/Su(H) molecules found in the asymmetric unit of the X-ray structure 5E24, in which one molecule is DNA-bound and the other is DNA-free (Yuan et al. 2016).

a) Structural alignment of DNA-bound (light blue) and DNA-free (yellow) forms of RBPJ/Su(H), which highlights the conformational differences between the two states.

b) Temperature-factor comparison of the DNA-bound and DNA-free states of RBPJ/Su(H), in which higher and lower temperature factors are colored in a gradient from red to blue. When compared to the DNA-bound state, the DNA-free state of RBPJ/Su(H) has higher temperature-factors within its DNA binding domains.

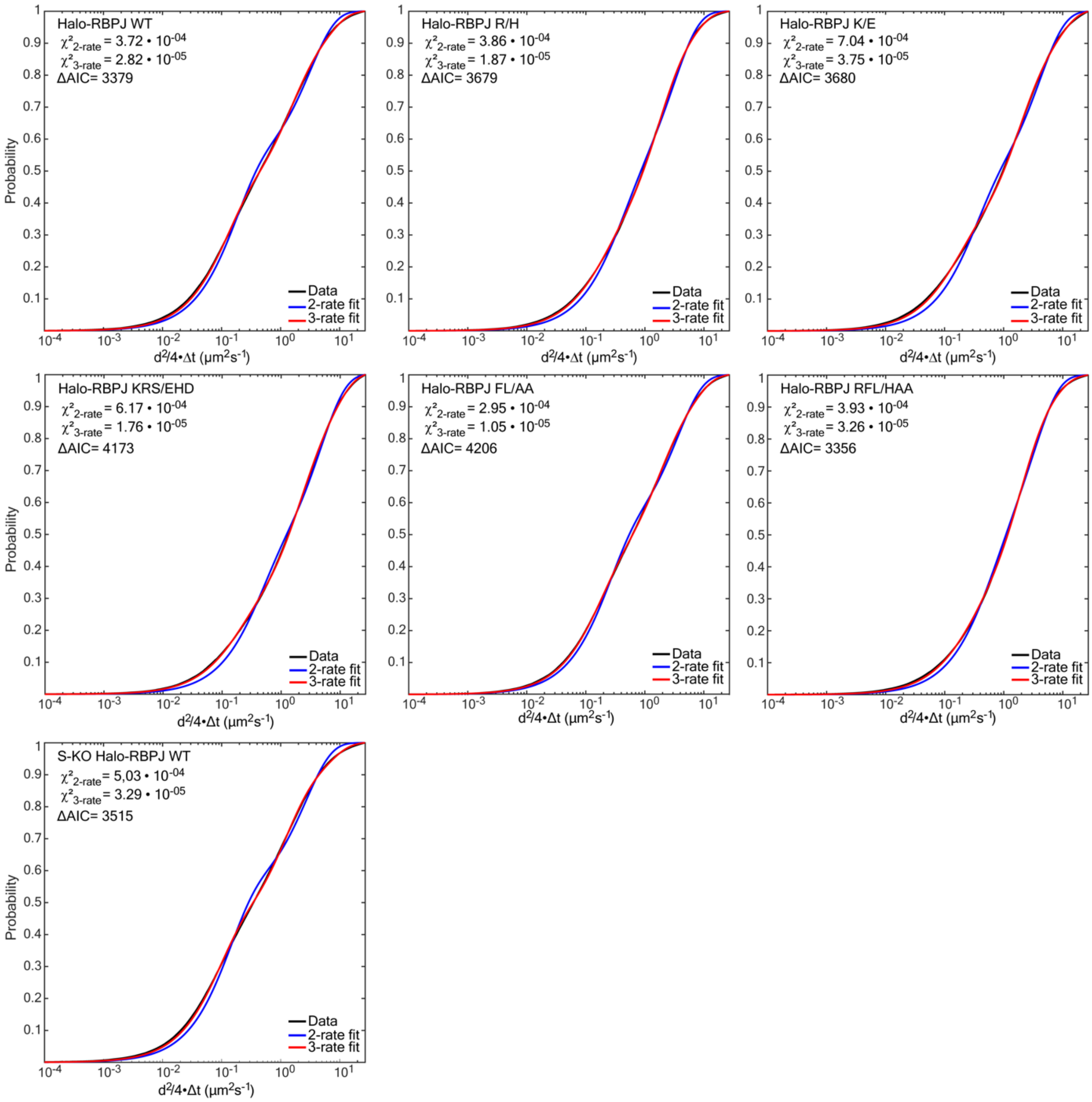

Supplementary Figure 12: Analysis of diffusion measurements

Cumulative jump distance distributions (black) of HT-RBPJ variants and fit of a two-component (blue) and a three-component diffusion model (Kuhn et al. 2022). According to the reduced χ^2 and the Akaike Information Criterion (AIC), the 3-component diffusion model described the data better. Experimental statistics are listed in Supplementary Table 5.

Source data are provided as Source Data file for Supplementary Figure 12.

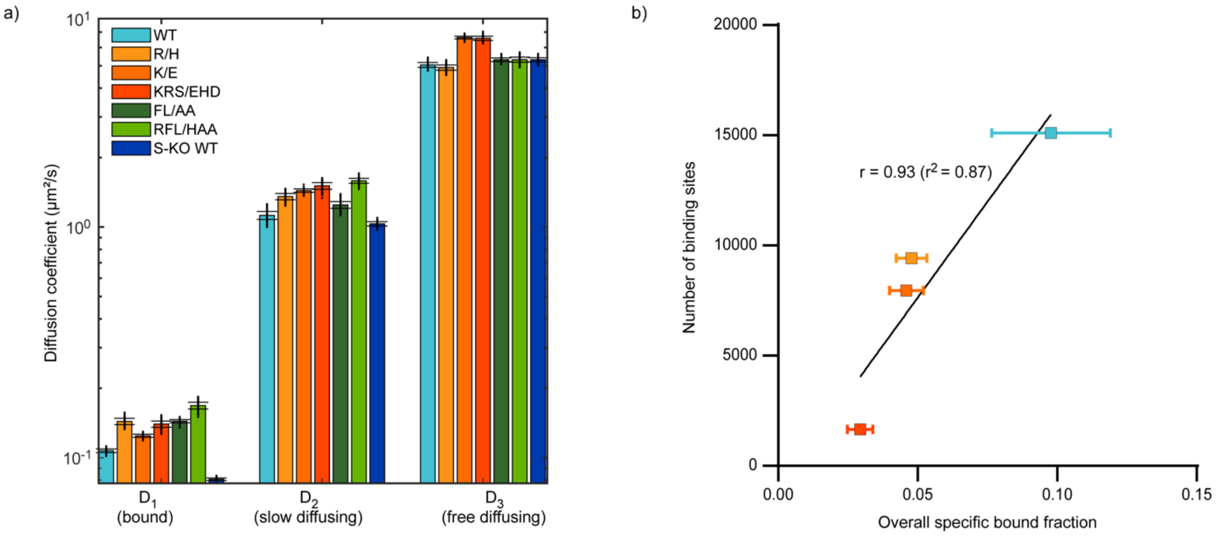

Supplementary Figure 13: Diffusion coefficients and bound fractions of HT-RBPJ variants

a) Diffusion coefficients of the three-component diffusion model and assignment to bound, slow and fast diffusing molecules. Data represents mean values ± s.d. from 400 resamplings with randomly selected 80 % of the data. Data are shown in Supplementary Table 4 and experimental statistics are listed in Supplementary Table 5.

b) Number of DNA binding sites found by ChIP-Seq versus overall specific bound fraction of HT-RBPJ variants. Values depict number of binding sites ± s.d (square root of value) from Fig. 1 and overall specific bound fraction ± s.d (Gaussian error propagation). Pearson's correlation coefficient calculated for HT-RBPJ-WT and DNA binding mutants. Color code as in a).

Source data are provided as Source Data file for Supplementary Figure 13a, b.

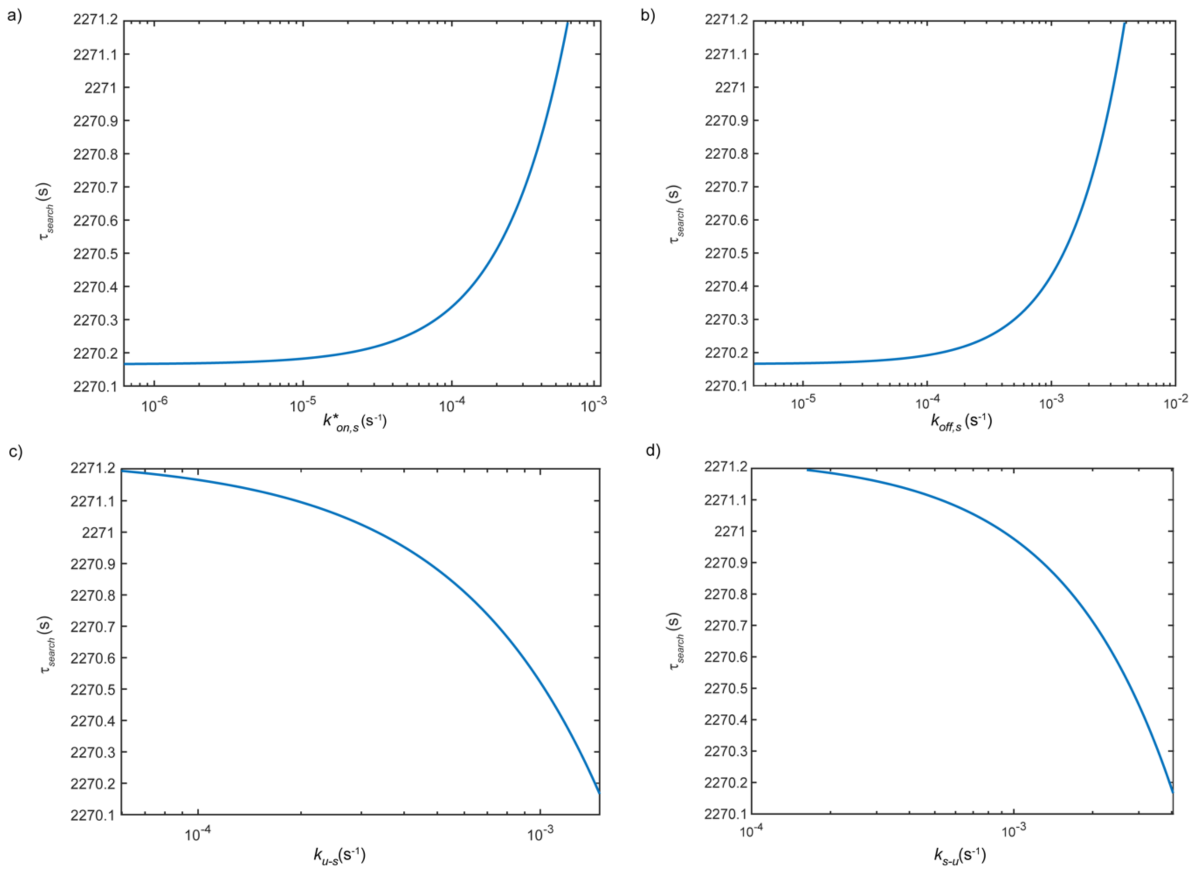

Supplementary Figure 14: Target site search time as function of various parameters.

a) Target site search time as function of direct association rate $k^*_{on,s}$.

b) Target site search time as function of direct dissociation rate $k_{off,s}$.

c) Target site search time as function microscopic association rate $k_{u\text{-}s}$.

d) Target site search time as function of microscopic dissociation rate $k_{s\text{-}u}$.

Source data are provided as Source Data file for Supplementary Figure 14.

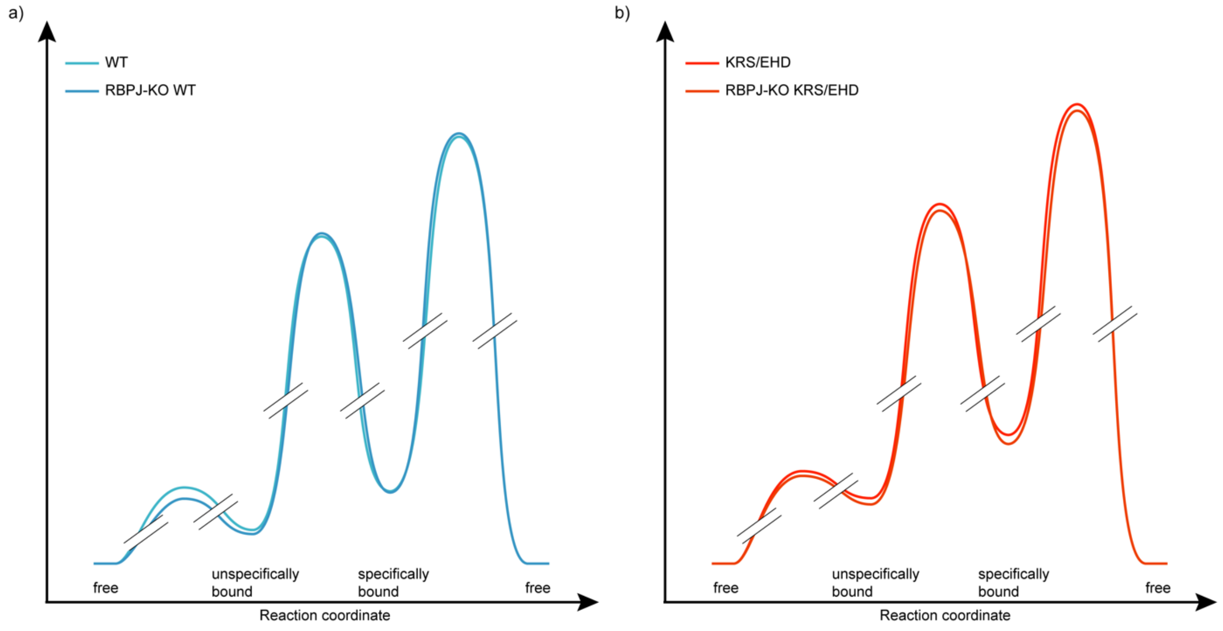

Supplementary Figure 15

a) and **b)** Binding energy landscape of RBPJ-WT and the triple DNA mutant KRS/EHD in Hela WT and in RBPJ knock out (#42).

Data are provided as Source Data file for Supplementary Figure 15.

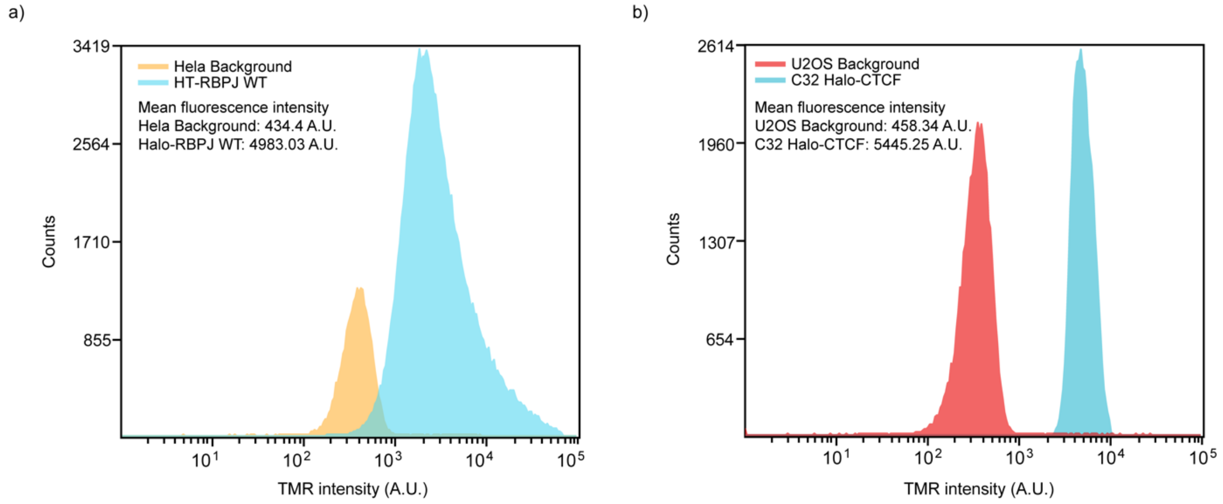

Supplementary Figure 16: Quantification of HT-RBPJ-WT molecule abundance

a) Flow cytometry intensity counts of HeLa cells stably expressing HT-RBPJ-WT labeled with HTL-TMR.

b) Flow cytometry intensity counts of U2OS C32 HT-CTCF cells labeled with HTL-TMR were used as reference (Cattoglio et al. 2019). Unstained HeLa or U2OS cells were used to estimate the background. Number of Events for HeLa unstained/Halo-RBPJ-WT TMR-stained: 25762/133444. Number of Events for U2OS unstained/ Halo-CTCF TMR-stained: 5927/38979.

Supplementary Tables

				Fractions of event spectrum		Fractions of state spectrum	
	Variant	k_d (s^{-1})	$k_{off,u}$ (s^{-1})	A_s^e (%)	A_u^e (%)	A_s^s (%)	A_u^s (%)
HeLa	HT-RBPJ-WT	0.00405 ± 0.0013	0.907 ± 0.04	0.17 ± 0.08	99.8 ± 0.1	26.7 ± 5.8	72.6 ± 6.7
	HT-RBPJ-R/H	0.0051 ± 0.001	1.42 ± 0.06	0.12 ± 0.02	99.9 ± 0.02	24.4 ± 4.3	70.4 ± 7.1
	HT-RBPJ-K/E	0.0063 ± 0.0006	1.28 ± 0.05	0.12 ± 0.02	99.9 ± 0.02	19.6 ± 2.6	80.0 ± 2.5
	HT-RBPJ-KRS/EHD	0.0076 ± 0.001	1.35 ± 0.03	0.11 ± 0.02	99.9 ± 0.02	16.3 ± 2.2	81.9 ± 5.0
	HT-RBPJ-FL/AA	0.0047 ± 0.0006	0.69 ± 0.04	0.60 ± 0.09	99.4 ± 0.1	45.6 ± 4.7	51.5 ± 4.4
	HT-RBPJ-RFL/HAA	0.007 ± 0.0006	1.01 ± 0.05	0.43 ± 0.06	99.6 ± 0.06	38.2 ± 2.9	61.8 ± 2.9
HeLa S-KO	HT-RBPJ-WT	0.0044 ± 0.001	0.6 ± 0.05	0.42 ± 0.11	99.6 ± 0.11	36.2 ± 5.3	62.7 ± 5.8
HeLa RBPJ-KO	HT-RBPJ-WT	0.0039 ± 0.0003	0.93 ± 0.03	0.20 ± 0.02	99.8 ± 0.019	31.0 ± 2.9	63.7 ± 3.8
HeLa RBPJ-KO	HT-RBPJ-KRS/EHD	0.00702 ± 0.0014	1.3 ± 0.054	0.115 ± 0.027	99.9 ± 0.027	16.9 ± 3.5	80.5 ± 5.8

Supplementary Table 1: Dissociation rates and amplitudes of state and event spectra obtained by GRID analysis of survival time distributions shown in Figure 2c and Supplementary Figure 9b.

	Variant	Specific residence time (τ_s) ± s.d. (s)	Unspecific residence time (τ_u) ± s.d. (s)
HeLa	Halo-RBPJ-WT	246.9 ± 79.3	1.1 ± 0.09
	Halo-RBPJ-R/H	194.2 ± 26.0	0.71 ± 0.02
	Halo-RBPJ-K/E	159.2 ± 14.7	0.78 ± 0.03
	Halo-RBPJ-KRS/EHD	133.5 ± 17.0	0.74 ± 0.02
	Halo-RBPJ-FL/AA	211.9 ± 25.9	1.43 ± 0.09
	Halo-RBPJ RFL/HAA	142.3 ± 11.7	0.99 ± 0.05
HeLa S-KO	Halo-RBPJ-WT	226.8 ± 50.9	1.67 ± 0.13
HeLa RBPJ-KO	HT-RBPJ-WT	277.8 ± 108.0	0.92 ± 0.08
HeLa RBPJ-KO	HT-RBPJ-KRS/EHD	142.45 ± 28.4	0.77 ± 0.03

Supplementary Table 2: Residence times of Halo-RBPJ variants at specific and unspecific sites, shown in Figure 2e,f and g.

		HeLa						HeLa S-KO	HeLa RBPJ-KO	HeLa RBPJ-KO
time-lapse condi-tion (s)		HT-RBPJ-WT	HT-RBPJ-R/H	HT-RBPJ-K/E	HT-RBPJ-KRS/EHD	HT-RBPJ-FL/AA	HT-RBPJ-RFL/HAA	HT-RBPJ-WT	HT-RBPJ-WT	HT-RBPJ-KRS/EHD
0.1	#movies	21	19	19	26	27	34	11	27	20
	#tracks	1459	1329	1039	1904	669	621	723	2167	1283
	#all events	19835	16990	17073	25937	14015	19846	9599	28193	13788
0.4	#movies	15	27	21	26	30	29	24	27	18
	#tracks	889	1064	1135	1669	731	402	981	2089	1013
	#all events	15220	20562	23255	26965	15747	17694	17988	34393	15373
1.6	#movies	28	24	14	26	23	37	14	31	17
	#tracks	2648	1978	1194	2711	1148	1024	974	4540	1431
	#all events	26782	22143	14678	31853	16386	20004	11724	48701	14117
6.4	#movies	27	20	20	18	27	24	19	18	24
	#tracks	1446	882	920	856	966	548	890	1603	864
	#all events	17315	11619	13110	12744	11931	9436	9617	1603	12410

Supplementary Table 3: Statistics of single-molecule time-lapse measurements.

	Variant	D_1 (µm^2/s)	D_2 (µm^2/s)	D_3 (µm^2/s)	A_1 (bound)	A_2 (slow diffusing)	A_3 (free diffusing)
HeLa	Halo-RBPJ-WT	0.107 ± 0.001	1.12 ± 0.02	5.0 ± 0.1	0.37 ± 0.01	0.35 ± 0.01	0.29 ± 0.01
	Halo-RBPJ-R/H	0.144 ± 0.002	1.36 ± 0.02	4.9 ± 0.1	0.20 ± 0.01	0.49 ± 0.01	0.31 ± 0.01
	Halo-RBPJ-K/E	0.125 ± 0.001	1.44 ± 0.01	6.6 ± 0.1	0.23 ± 0.01	0.44 ± 0.01	0.33 ± 0.01
	Halo-RBPJ-KRS/EHD	0.140 ± 0.002	1.51 ± 0.01	6.6 ± 0.1	0.18 ± 0.01	0.40 ± 0.01	0.41 ± 0.01
	Halo-RBPJ-FL/AA	0.144 ± 0.001	1.24 ± 0.02	5.3 ± 0.1	0.33 ± 0.01	0.35 ± 0.01	0.32 ± 0.01
	Halo-RBPJ-RFL/HAA	0.169 ± 0.003	1.59 ± 0.02	5.3 ± 0.1	0.15 ± 0.01	0.56 ± 0.01	0.29 ± 0.01
HeLa S-KO	Halo-RBPJ-WT	0.081 ± 0.001	1.03 ± 0.01	5.3 ± 0.1	0.38 ± 0.01	0.40 ± 0.01	0.22 ± 0.01
Hela RBPJ-KO	HT-RBPJ-WT	0.124 ± 0.001	1.21 ± 0.01	6.2 ± 0.03	0.39 ± 0.01	0.40 ± 0.01	0.22 ± 0.01
Hela RBPJ-KO	HT-RBPJ-KRS/EHD	0.1 ± 0.01	1.22 ± 0.01	6.2 ± 0.03	0.21 ± 0.01	0.43 ± 0.02	0.36 ± 0.01

Supplementary Table 4: Diffusion coefficients and fractions obtained from cumulative jump distance distributions shown in Figure 3c and Supplementary Figure 13a.

	Variant	Days	#Cells	#Tracks	#all events
**HeLa**	**Halo-RBPJ-WT**	4	9	25204	57546
	**Halo-RBPJ-R/H**	2	12	26237	55238
	**Halo-RBPJ-K/E**	2	7	77936	174652
	**Halo-RBPJ-KRS/EHD**	2	11	27630	60785
	**Halo-RBPJ-FL/AA**	3	19	33548	77632
	**Halo-RBPJ-RFL/HAA**	3	22	55242	110146
**HeLa S-KO**	**Halo-RBPJ-WT**	1	13	42339	100670
**Hela RBPJ-KO**	**HT-RBPJ WT**	2	25	167314	366421
**Hela RBPJ-KO**	**HT-RBPJ-KRS**	2	24	147086	315285

Supplementary Table 5: Statistics of continuous movies for diffusion analysis.

	$k_{on,u}$ (s^{-1})	$k_{u\text{-}s}$ (s^{-1})	$k_{s\text{-}u}$ (s^{-1})	$k_{off,s}$ (s^{-1})	$k_{on,s}$ (s^{-1})	τ_{search} (s)
**Max_τ_{search}**	0.3806 ± 0.0813	0.0004 ± 0.0001	0.0012 ± 0.0006	0.0029	0.0004 ± 0.0002	2270.9 ± 616.5
**Min_τ_{search}**	0.3806 ± 0.0813	0.0015 ± 0.0003	0.0041 ± 0.0019	$4.05*10^{-6}$	$6.25*10^{-7}$ ± $2.73*10^{-7}$	2270.1 ± 582.0

Supplementary Table 6: Limits of the parameters of the three-state model of facilitated diffusion determined for HT-RBPJ-WT. Errors were calculated based on Gaussian error propagation. $k_{off,s}$ was fixed relative to k_d.

	Variant	p_s	p_u	p_f	$k_{on,u}$ (s^{-1})	$k_{off,u}$ (s^{-1})	$k_{u\text{-}s}$ (s^{-1})	$k_{s\text{-}u}$ (s^{-1})	k_d (s^{-1})	$k_{off,s}$ (s^{-1})	$k_{on,s}$ (s^{-1})	τ_{search} (s)
**HeLa**	**HT-RBPJ-WT**	0.10 ± 0.02	0.27 ± 0.03	0.63 ± 0.01	0.3806 ± 0.0813	0.907 ± 0.07	0.0013 ± 0.0003	0.0038 ± 0.0018	0.0041 ± 0.0013	0.000286	0.0000441 ± 0.0000166	2270.2 ± 538.3
	**HT-RBPJ-R/H**	0.05 ± 0.01	0.14 ± 0.01	0.80 ± 0.01	0.2639 ± 0.0696	1.41 ± 0.04	0.0015 ±0.0003	0.0048 ± 0.0027	0.0052 ± 0.001	0.000363	0.0000217 ± 0.0000100	3854.3 ± 1073.5
	**HT-RBPJ-K/E**	0.05 ± 0.01	0.19 ± 0.01	0.77 ± 0.01	0.3139 ± 0.0493	1.28 ± 0.05	0.0014 ± 0.0003	0.0058 ± 0.0029	0.0063 ± 0.0006	0.000443	0.0000266 ± 0.0000115	3299.8 ± 733.3
	**HT-RBPJ-KRS/EHD**	0.03 ± 0.01	0.15 ± 0.01	0.82 ± 0.01	0.2491 ± 0.0803	1.35 ± 0.04	0.0014 ± 0.0003	0.007 ± 0.0055	0.0075 ± 0.001	0.000528	0.0000190 ± 0.0000133	4391.5 ± 1385.7
	**HT-RBPJ-FL/AA**	0.15 ± 0.02	0.17 ± 0.01	0.67 ± 0.01	0.1786 ± 0.0373	0.70 ± 0.04	0.0039 ± 0.0008	0.0044 ± 0.0016	0.0047 ± 0.0006	0.000333	0.0000763 ± 0.0000147	1159.6 ± 282.7
	**HT-RBPJ-RFL/HAA**	0.06 ± 0.01	0.09 ± 0.01	0.85 ± 0.01	0.1120 ± 0.0446	1.01 ± 0.05	0.0041 ± 0.0008	0.0066 ± 0.0041	0.007 ± 0.0006	0.000496	0.0000340 ± 0.0000148	2305.0 ± 881.2
**HeLa S-KO**	**HT-RBPJ-WT**	0.14 ± 0.02	0.24 ± 0.02	0.62 ± 0.01	0.2289 ± 0.0444	0.6 ± 0.05	0.0024 ± 0.0005	0.0041 ± 0.0015	0.0044 ± 0.001	0.000311	0.0000691 ± 0.0000156	1408.5 ± 324.3
**Hela RBPJ -KO**	**HT-RBPJ-WT**	0.10 ± 0.02	0.29 ± 0.03	0.61 ± 0.01	0.5086 ± 0.1098	1.09 ± 0.10	0.0012 ± 0.0002	0.0034 ± 0.0017	0.0036 ± 0.0014	0.000254	0.0000405 ± 0.0000160	2552.9 ± 602.3
**Hela RBPJ -KO**	**HT-RBPJ-KRS/EHD**	0.04 ± 0.01	0.17 ± 0.01	0.79 ± 0.01	0.2815 ± 0.0857	1.3 ± 0.05	0.0014 ± 0.0003	0.0065 ± 0.0056	0.0070 ± 0.0014	0.000495	0.0000225 ± 0.0000173	3810.8 ± 1147.0

Supplementary Table 7: Overall fractions, kinetic rates and target site search time for RBPJ variants shown in Figure 3e. Errors were calculated based on Gaussian error propagation. Error for $k_{u\text{-}s}$ was estimated with 20% of the value. $k_{off,s}$ was fixed relative to k_d.

	Variant	$\Delta G^{\#}_{f\text{->}u}$ (k_BT)	ΔG_u (k_BT)	$\Delta G^{\#}_{u\text{->}s}$ (k_BT)	ΔG_s (k_BT)	$\Delta G^{\#}_{f\text{->}s}$ (k_BT)
**HeLa**	**Halo-RBPJ-WT**	0.97 ± 0.21	0.87 ± 0.23	7.45 ± 1.49	1.87 ± 0.38	10.03 ± 0.38
	**Halo-RBPJ-R/H**	1.33 ± 0.26	1.68 ± 0.27	8.16 ± 1.63	2.82 ± 0.46	10.74 ± 0.46
	**Halo-RBPJ-K/E**	1.16 ± 0.16	1.41± 0.16	7.96 ± 1.59	2.81 ± 0.43	10.53 ± 0.43
	**Halo-RBPJ-KRS/EHD**	1.39 ± 0.32	1.69 ± 0.32	8.29 ± 1.66	3.32 ± 0.70	10.87 ± 0.70
	**Halo-RBPJ-FL/AA**	1.72 ± 0.21	1.36 ± 0.22	6.90 ± 1.38	1.47 ± 0.19	9.48 ± 0.19
	**Halo-RBPJ-RFL/HAA**	2.19 ± 0.40	2.20 ± 0.40	7.71 ±1.54	2.68 ± 0.44	10.29 ± 0.44
**HeLa S-KO**	**Halo-RBPJ-WT**	1.48 ± 0.20	0.96 ± 0.21	7.00 ±1.40	1.50 ± 0.23	9.58 ± 0.24
**Hela RBPJ-KO**	**HT-RBPJ-WT**	0.68 ± 0.22	0.76 ± 0.23	7.54 ± 1.51	1.84 ± 0.40	10.12 ± 0.40
**Hela RBPJ-KO**	**HT-RBPJ-KRS/EHD**	1.27 ± 0.31	1.53 ± 0.31	8.12 ± 1.62	3.09 ± 0.77	10.70 ± 0.77

Supplementary Table 8: Free energy differences between the free state and transition or bound states of HT-RBPJ variants shown in Figure 4d. Errors were calculated based on Gaussian error propagation.

	Variant	$\Delta G^{\#}_{f\text{->}u}$ (k_BT)	ΔG_u (k_BT)	$\Delta G^{\#}_{u\text{->}s}$ (k_BT)	ΔG_s (k_BT)	$\Delta G^{\#}_{f\text{->}s}$ (k_BT)
**U2OS**	**TALE 9R**	0.55 ± 0.40	0.42 ± 0.41	7.45 ± 1.49	1.90 ± 0.45	9.97 ± 0.45
	**TALE 13R**	0.97 ± 0.32	0.78 ± 0.32	6.46 ± 1.29	0.82 ± 0.34	8.98 ± 0.34
	**TALE 15R**	1.07 ± 0.33	0.69 ± 0.34	7.05 ± 1.41	0.96 ± 0.30	9.56 ± 0.30
	**TALE 19R**	0.41 ± 0.27	0.61 ± 0.28	6.80 ± 1.36	1.50 ± 0.40	9.32 ± 0.40

Supplementary Table 9: Free energy differences between the free state and transition or bound states of TALE-TFs shown in Figure 4e. Errors were calculated based on Gaussian error propagation.

	Variant	$\Delta G^{\#}_{f\text{->}u}$ (k_BT)	ΔG_u (k_BT)	$\Delta G^{\#}_{f\text{->}s}$ (k_BT)	ΔG_s (k_BT)
**Embryonic stem cells**	**SOX2**	1.30 ± 0.21	1.51 ± 0.15	5.93 ± 0.05	3.44 ± 0.16
	**SOX2D**	0.84 ± 0.17	1.04 ± 0.21	5.14 ± 0.09	2.98 ± 0.15
	**SOX2M**	1.72 ± 0.17	1.97 ± 0.22	6.40 ± 0.09	4.17 ± 0.23
**Hemocytes**	**GAF**	0.95	-0.36	5.00	0.14
	**GAF dPOZ**	0.28	-0.62	4.65	0.89
	**GAF dQ**	0.54	-0.53	4.35	0.46

Supplementary Table 10: Free energy differences between the free state and transition or bound states shown in Figure 4f and 4g calculated from published kinetic rates. Errors were calculated based on Gaussian error propagation.

Plasmids	Description
pGa981-6	Reporter plasmid containing twelve RBPJ binding sites, a Beta-Globin promoter as well as the gene for Luciferase
pcDNA3.1(-)hsNICD	Expression plasmid for human Notch1 Intracellular domain
pcDNA3.1-Flag-mRBPJ-CRr(wt)	Expression plasmid for murine Flag-tagged RBPJ wildtype
pcDNA3.1-Flag-mRBPJ-CRr(R218H)	Expression plasmid for murine Flag-tagged RBPJ R218H
pcDNA3.1-Flag-mRBPJ-CRr(K195E)	Expression plasmid for murine Flag-tagged RBPJ K195E
pcDNA3.1-Flag-mRBPJ-CRr(KRS)	Expression plasmid for murine Flag-tagged RBPJ K195E/R218H/S221D
pcDNA3.1-Flag-mRBPJ-CRr(F261A/L388A)	Expression plasmid for murine Flag-tagged RBPJ F261A/L388A
pcDNA3.1-Flag-mRBPJ-CRr(R218H/F261A/L388A)	Expression plasmid for murine Flag-tagged RBPJ R218H/F261A/L388A
pcDNA3.1-Flag-mRBPJ-CRr-VP16(wt)	Expression plasmid for murine Flag-tagged and VP16-fused RBPJ wildtype
pcDNA3.1-Flag-mRBPJ-CRr-VP16(R218H)	Expression plasmid for murine Flag-tagged and VP16-fused RBPJ R218H
pcDNA3.1-Flag-mRBPJ-CRr-VP16(K195E)	Expression plasmid for murine Flag-tagged and VP16-fused RBPJ K195E
pcDNA3.1-Flag-mRBPJ-CRr-VP16(KRS)	Expression plasmid for murine Flag-tagged and VP16-fused RBPJ KRS
pcDNA3.1-Flag-mRBPJ-CRr-VP16(F261A/L388A)	Expression plasmid for murine Flag-tagged and VP16-fused RBPJ F261A/L388A
pcDNA3.1-Flag-mRBPJ-CRr-VP16(R218H/F261A/L388A)	Expression plasmid for murine Flag-tagged and VP16-fused RBPJ R218H/F261A/L388A

Supplementary Table 11: List of plasmids used in luciferase assays shown in Figure 1b and 1c.

Plasmids	Description
pLVTO-Halo-mRBPJ-CRr(wt)	Expression plasmid for murine Halo-tagged RBPJ wildtype
pLVTO-Halo-mRBPJ-CRr(KRS)	Expression plasmid for murine Halo-tagged RBPJ K195E/R218H/S221D
pLVTO-Halo-mRBPJ-CRr(F261A/L388A)	Expression plasmid for murine Halo-tagged F261A/L388A
pLVTO-Halo-mRBPJ-CRr(R218H/F261A/L388A)	Expression plasmid for murine Halo-tagged R218H/F261A/L388A
pcDNA3.1(-)Flag1-hsNICD	Expression plasmid for human Flag-tagged Notch1 Intracellular domain
pcDNA3-Flag-SHARP(2770-3127)	Expression plasmid for human Flag-tagged RBPJ binding domain of-SHARP.

Supplementary Table 12: List of plasmids used in immunoprecipitation shown in Figure S2a,b,c,d and in luciferase assay in S2g.

CRISPR/Cas9 guides	
hRBPJ guide #1	5’- TCA TGC CAG TTC ACA GCA GT -3’
hRBPJ guide #2	5’- TCC TTC TAC ATG CAA GTA TC -3’
hSHARP guide #1	5’- GCA ACT TAC CCG AGA ACG TG -3’
hSHARP guide #2	5´- TCC CGG TGA ATC AAA GAC GG -3’

Supplementary Table 13: Guide sequences used for CRISPR/Cas9 experiments to generate knock-out cell lines.

Gene	Primer	Gene Globe ID
ACTIN	Hs_ACTB_2_SG QuantiTect Primer Assay	QT01680476
HES1	Hs_HES1_1_SG QuantiTect Primer Assay	QT00039648
HEY1	Hs_HEY1_1_SG QuantiTect Primer Assay	QT00035644
HEY2	Hs_HEY2_3_SG QuantiTect Primer Assay	QT01849701
SHARP	Hs_SPEN_1_SG QuantiTect Primer Assay	QT00042196

Supplementary Table 14: Primers used for real time qPCR.

Antibody	Catalogue Nr.:	Company	Application	Type
anti-RBPJ	5313	Cell Signaling Technology	mix 20 µL Ab with 500 µg chromatin	monoclonal (rabbit)
Anti-His2Av	61686	Active Motif	2 µg/500 ng chromatin	N.A.

Supplementary Table 15: Antibody used in ChIP-seq experiments

Primary Antibody	Catalogue Nr.:	Company	Type	Dilution	Suppl. Figure
Anti-RBPJ (T6709)	IIM-2ARBP2	Cosmo Bio Co., Ltd.	Monoclonal (Rat)	1:2000	S2a,b,c,d S3b, S4g, S8b
Anti-Beta-ACTIN (A1978)	A1978	Sigma-Aldrich	Monoclonal (Mouse)	1:5000	S3b, S4g, S4h, S8b
Anti-Flag (M5)	F4042	Sigma-Aldrich	Monoclonal (Mouse)	1:10000	S2a,b,c,d
Anti-SHARP.1	-	-	Polyclonal anti-serum (Rabbit)	1:1000	S4b
Anti-Halo-Tag (G921A)	G9211	Promega GmbH	Monoclonal (Mouse)	1:500	S4h
**Secondary Antibody**	**Catalogue Nr.:**	**Company**	**Type**	**Dilution**	**Suppl. Figure**
Peroxidase AffiniPure Anti-rat IgG HRP-conjugated	112-035-071	Jackson Im-muno Research	Whole antibody	1:5000	S2a,b,c,d, S3b, S4g, S8b
ECL™ Anti-mouse IgG HRP-conjugated	NA931V	Cytiva	Whole antibody	1:5000	S2a,b,c,d, S3b, S4g,h, S8b
ECL™ Anti-Rabbit IgG HRP-conjugated	NA934V	Cytiva	Whole antibody	1:5000	S4b

Supplementary Table 16: List of antibodies used for Western Blots.

Primary Antibody	Catalogue Nr.:	Company	Type	Dilution	Suppl. Figure
Anti-SHARP.1	-	-	Polyclonal rabbit antiserum	1:500	S4c
**Secondary Antibody**	**Catalogue Nr.:**	**Company**	**Type**	**Dilution**	**Suppl. Figure**
Goat anti-Rabbit IgG (H+L) Cross-Adsorbed secondary Antibody, Alexa Fluor™ 488	A11008	Invitrogen	Polyclonal	1:1000	S4c

Supplementary Table 17: List of antibodies used for immunofluorescence microscopy.
