# Supplementary material for: Effective in vivo binding energy landscape illustrates kinetic stability of RBPJ-DNA binding
Source: Nat Commun. 2025 Feb 1;16:1259. doi: 10.1038/s41467-025-56515-4 (PMC11787368; doi:10.1038/s41467-025-56515-4)
Supplement: Supplementary file 2 — Reporting Summary [file 41467_2025_56515_MOESM2_ESM.pdf]

nature portfolio

Corresponding author(s): Franz Oswald and J. Christof M. Gebhardt

Last updated by author(s): Jan 10, 2025

Reporting Summary

Nature Portfolio wishes to improve the reproducibility of the work that we publish. This form provides structure for consistency and transparency in reporting. For further information on Nature Portfolio policies, see our Editorial Policies and the Editorial Policy Checklist.

Statistics

For all statistical analyses, confirm that the following items are present in the figure legend, table legend, main text, or Methods section.

n/a	Confirmed	
☐	☒	The exact sample size (*n*) for each experimental group/condition, given as a discrete number and unit of measurement
☐	☒	A statement on whether measurements were taken from distinct samples or whether the same sample was measured repeatedly
☐	☒	The statistical test(s) used AND whether they are one- or two-sided<br>*Only common tests should be described solely by name; describe more complex techniques in the Methods section.*
☒	☐	A description of all covariates tested
☒	☐	A description of any assumptions or corrections, such as tests of normality and adjustment for multiple comparisons
☐	☒	A full description of the statistical parameters including central tendency (e.g. means) or other basic estimates (e.g. regression coefficient) AND variation (e.g. standard deviation) or associated estimates of uncertainty (e.g. confidence intervals)
☐	☒	For null hypothesis testing, the test statistic (e.g. *F*, *t*, *r*) with confidence intervals, effect sizes, degrees of freedom and *P* value noted<br>*Give P values as exact values whenever suitable.*
☒	☐	For Bayesian analysis, information on the choice of priors and Markov chain Monte Carlo settings
☒	☐	For hierarchical and complex designs, identification of the appropriate level for tests and full reporting of outcomes
☐	☒	Estimates of effect sizes (e.g. Cohen's *d*, Pearson's *r*), indicating how they were calculated

Our web collection on statistics for biologists contains articles on many of the points above.

Software and code

Policy information about availability of computer code

Data collection	Single-molecule microscopy movies were acquired using Nikon NIS-Elements Version 4.40.00 64 bit.
Data analysis	Single-molecule tracking and analysis was performed in Matlab R2022a with the TrackIT Software (Kuhn et al. Sci Rep 11, 9465 (2021), https://gitlab.com/GebhardtLab/TrackIt, https://doi.org/10.5281/zenodo.7092296).<br>Pearson's correlation analysis was performed in GraphPad Prism 9.5.1.

For manuscripts utilizing custom algorithms or software that are central to the research but not yet described in published literature, software must be made available to editors and reviewers. We strongly encourage code deposition in a community repository (e.g. GitHub). See the Nature Portfolio guidelines for submitting code & software for further information.

Data

Policy information about availability of data

All manuscripts must include a data availability statement. This statement should provide the following information, where applicable:

- Accession codes, unique identifiers, or web links for publicly available datasets
- A description of any restrictions on data availability
- For clinical datasets or third party data, please ensure that the statement adheres to our policy

Source data for figures and gels are provided as a separate Source Data file. ChIP-Seq data was uploaded to the Gene Expression Omnibus repository (GEO accession number: GSE249973 [https://www.ncbi.nlm.nih.gov/geo/query/acc.cgi?acc=GSE249973]). Single-particle tracking data are freely available at Data Dryad [https://

doi.org/10.5061/dryad.mkkwh716k]84. Data supporting the findings of this manuscript will be additionally available from the corresponding authors upon reasonable request.

Research involving human participants, their data, or biological material

Policy information about studies with human participants or human data. See also policy information about sex, gender (identity/presentation), and sexual orientation and race, ethnicity and racism.

Reporting on sex and gender	*Use the terms sex (biological attribute) and gender (shaped by social and cultural circumstances) carefully in order to avoid confusing both terms. Indicate if findings apply to only one sex or gender; describe whether sex and gender were considered in study design; whether sex and/or gender was determined based on self-reporting or assigned and methods used. Provide in the source data disaggregated sex and gender data, where this information has been collected, and if consent has been obtained for sharing of individual-level data; provide overall numbers in this Reporting Summary. Please state if this information has not been collected. Report sex- and gender-based analyses where performed, justify reasons for lack of sex- and gender-based analysis.*
Reporting on race, ethnicity, or other socially relevant groupings	*Please specify the socially constructed or socially relevant categorization variable(s) used in your manuscript and explain why they were used. Please note that such variables should not be used as proxies for other socially constructed/relevant variables (for example, race or ethnicity should not be used as a proxy for socioeconomic status). Provide clear definitions of the relevant terms used, how they were provided (by the participants/respondents, the researchers, or third parties), and the method(s) used to classify people into the different categories (e.g. self-report, census or administrative data, social media data, etc.) Please provide details about how you controlled for confounding variables in your analyses.*
Population characteristics	*Describe the covariate-relevant population characteristics of the human research participants (e.g. age, genotypic information, past and current diagnosis and treatment categories). If you filled out the behavioural & social sciences study design questions and have nothing to add here, write "See above."*
Recruitment	*Describe how participants were recruited. Outline any potential self-selection bias or other biases that may be present and how these are likely to impact results.*
Ethics oversight	*Identify the organization(s) that approved the study protocol.*

Note that full information on the approval of the study protocol must also be provided in the manuscript.

Field-specific reporting

Please select the one below that is the best fit for your research. If you are not sure, read the appropriate sections before making your selection.

- [x] Life sciences
- [ ] Behavioural & social sciences
- [ ] Ecological, evolutionary & environmental sciences

For a reference copy of the document with all sections, see nature.com/documents/nr-reporting-summary-flat.pdf

Life sciences study design

All studies must disclose on these points even when the disclosure is negative.

Sample size	The number of movies and cells for singe-molecule tracking is given in Supplementary Table 3 and 5. For time-lapse measurements at least 50 different cells were measured for each condition and obtained from several different measurement days. In case of diffusion analysis we measured a minimum of 5 different cells.
Data exclusions	Strongly drifting cells were excluded from analysis. Cells showing high expression level and having more than 20 single molecule detections in a frame were excluded from analysis to prevent wrongly connected tracks.
Replication	Time-lapse measurements were performed on several cells at different days with a minimum of 50 cells per condition. Continuous 11.7 ms movies were taken from minimum 5 different cells. Replica measurements were performed successfully.
Randomization	On measurement days we selected random cells for recording single-molecule movies. Cells within a dish stably express the same proteins and replicates where measured at different days. Hence, there was no randomizantion required to exclude bias.
Blinding	Blinding was not required in this study. To exclude bias, we recorded and analyzed all data under the same experimental conditions.

Reporting for specific materials, systems and methods

We require information from authors about some types of materials, experimental systems and methods used in many studies. Here, indicate whether each material, system or method listed is relevant to your study. If you are not sure if a list item applies to your research, read the appropriate section before selecting a response.

Materials & experimental systems

n/a	Involved in the study
☐	☒ Antibodies
☐	☒ Eukaryotic cell lines
☒	☐ Palaeontology and archaeology
☒	☐ Animals and other organisms
☒	☐ Clinical data
☒	☐ Dual use research of concern
☒	☐ Plants

Methods

n/a	Involved in the study
☐	☒ ChIP-seq
☐	☒ Flow cytometry
☒	☐ MRI-based neuroimaging

Antibodies

Antibodies used

Information regarding the antibodies used in this study are shown in Supplementary Tables 15-17 and are described in method sections ChIP-Seq, Western Blotting, and widefield fluorescence microscopy

Validation

Anti-SHARP.1:
Validation of the SHARP.1 antibody was done before by fluorescence imaging of a SHARP knock-out cell line.
Reference: SHARP is a novel component of the Notch/RBP-Jκ signalling pathway" Oswald et al, EMBO J. 21,5417 (2002), https://doi.org/10.1093/emboj/cdf549
We further validated anti-SHARP.1 in this work by Western Blotting using Sharp-KO cell lines.
Anti-RBPJ T6709:
Manufacturer website provides validation statements and relevant citations
URL: https://www.cosmobiousa.com/products/anti-rbp-jk-mab-clone-t6709
Anti-RBPJ 5313S:
Manufacturer website provides validation statements and relevant citations:
https://www.cellsignal.com/products/primary-antibodies/rbpsuh-d10a4-xp-rabbit-mab/5313?srsltid=AfmBOorQb407REeRRtAVGyqpMcJeSv3Q44Py3r_d__n7ciX5Kdm_nlpZ
Anti-Beta-ACTIN:
Manufacturer website provides validation statements and relevant citations
URL: https://www.sigmaaldrich.com/DE/en/product/sigma/a1978
Anti-Halo-Tag:
Manufacturer website provides validation statements and relevant citations
URL: https://www.promega.de/products/protein-detection/primary-and-secondary-antibodies/anti-halotag-monoclonal-antibody/?catNum=G9211#specifications
Anti-Flag (M5) F4042:
Manufacturer website provides validation statements and relevant citations
https://www.sigmaaldrich.com/DE/de/product/sigma/f4042

Eukaryotic cell lines

Policy information about cell lines and Sex and Gender in Research

Cell line source(s)

HeLa (ATCC CCL-2), HEK293 (ATCC: CRL-1573)
LentiX 293T cells for lentiviral packaging, obtained from Clonetech Labratories Inc.

Authentication

Information regarding authentication is provided by American Type Culture Collection (ATCC)

Mycoplasma contamination

None. Mycoplasma tests were performed on a monthly basis for all used cell lines and all cell lines were tested negative.

Commonly misidentified lines
(See ICLAC register)

no commonly mis-identified cells have been used in this study.

Plants

Seed stocks

Report on the source of all seed stocks or other plant material used. If applicable, state the seed stock centre and catalogue number. If plant specimens were collected from the field, describe the collection location, date and sampling procedures.

Novel plant genotypes

Describe the methods by which all novel plant genotypes were produced. This includes those generated by transgenic approaches, gene editing, chemical/radiation-based mutagenesis and hybridization. For transgenic lines, describe the transformation method, the number of independent lines analyzed and the generation upon which experiments were performed. For gene-edited lines, describe the editor used, the endogenous sequence targeted for editing, the targeting guide RNA sequence (if applicable) and how the editor was applied.

Authentication

Describe any authentication procedures for each seed stock used or novel genotype generated. Describe any experiments used to assess the effect of a mutation and, where applicable, how potential secondary effects (e.g. second site T-DNA insertions, mosiacism, off-target gene editing) were examined.

ChIP-seq

Data deposition

☒ Confirm that both raw and final processed data have been deposited in a public database such as GEO.

☒ Confirm that you have deposited or provided access to graph files (e.g. BED files) for the called peaks.

Data access links<br>*May remain private before publication.*	GEO accession: GSE249973
Files in database submission	Raw sequencing reads (FASTQ), normalized coverage tracks (.bigwig) , Peak coordinates (.bed):<br><br>GSM7968292<br>Input_KO_42<br>GSM7968293<br>Input_KO_44<br>GSM7968294<br>Input_RBPJ_KO_42_Halo<br>GSM7968295<br>Input_RBPJ_KO_42_HaloRBPJ_FL_AA<br>GSM7968296<br>Input_RBPJ_KO_42_HaloRBPJ_K195E<br>GSM7968297<br>Input_RBPJ_KO_42_HaloRBPJ_KRS<br>GSM7968298<br>Input_RBPJ_KO_42_HaloRBPJ_R218H<br>GSM7968299<br>Input_RBPJ_KO_42_HaloRBPJ_RFL_HAA<br>GSM7968300<br>Input_RBPJ_KO_42_HaloRBPJ_WT<br>GSM7968301<br>Input_RBPJ_KO_eV<br>GSM7968302<br>Input_SHARP_KO_30<br>GSM7968303<br>Input_SHARP_KO_30_Halo_only_1<br>GSM7968304<br>Input_SHARP_KO_30_HaloRBPJ_WT_1<br>GSM7968305<br>Input_SHARP_KO_36<br>GSM7968306<br>Input_SHARP_KO_36_Halo_only_2<br>GSM7968307<br>Input_SHARP_KO_36_HaloRBPJ_WT_2<br>GSM7968308<br>Input_SHARP_KO_eV<br>GSM7968309<br>RBPJ_KO_42_1<br>GSM7968310<br>RBPJ_KO_42_2<br>GSM7968311<br>RBPJ_KO_42_Halo_1<br>GSM7968312<br>RBPJ_KO_42_Halo_2<br>GSM7968313<br>RBPJ_KO_42_HaloRBPJ_FL_AA_1<br>GSM7968314<br>RBPJ_KO_42_HaloRBPJ_FL_AA_2<br>GSM7968315<br>RBPJ_KO_42_HaloRBPJ_K195E_1<br>GSM7968316<br>RBPJ_KO_42_HaloRBPJ_K195E_2<br>GSM7968317<br>RBPJ_KO_42_HaloRBPJ_KRS_1<br>GSM7968318<br>RBPJ_KO_42_HaloRBPJ_KRS_2<br>GSM7968319<br>RBPJ_KO_42_HaloRBPJ_R218H_1

GSM7968320
RBPJ_KO_42_HaloRBPJ_R218H_2
GSM7968321
RBPJ_KO_42_HaloRBPJ_RFL_HAA_1
GSM7968322
RBPJ_KO_42_HaloRBPJ_RFL_HAA_2
GSM7968323
RBPJ_KO_42_HaloRBPJ_WT_1
GSM7968324
RBPJ_KO_42_HaloRBPJ_WT_2
GSM7968325
RBPJ_KO_44_1
GSM7968326
RBPJ_KO_44_2
GSM7968327
RBPJ_KO_eV_1
GSM7968328
RBPJ_KO_eV_2
GSM7968329
SHARP_KO_30_1
GSM7968330
SHARP_KO_30_2
GSM7968331
SHARP_KO_30_Halo_only_1
GSM7968332
SHARP_KO_30_Halo_only_2
GSM7968333
SHARP_KO_30_HaloRBPJ_WT_1
GSM7968334
SHARP_KO_30_HaloRBPJ_WT_2
GSM7968335
SHARP_KO_36_1
GSM7968336
SHARP_KO_36_2
GSM7968337
SHARP_KO_36_Halo_only_1
GSM7968338
SHARP_KO_36_Halo_only_2
GSM7968339
SHARP_KO_36_HaloRBPJ_WT_1
GSM7968340
SHARP_KO_36_HaloRBPJ_WT_2
GSM7968341
SHARP_KO_eV_1
GSM7968342
SHARP_KO_eV_2
GSM7968343
Input_SHARP_KO_eV_for_36
GSM7968344
SHARP_KO_eV_for_36_1
GSM7968345
SHARP_KO_eV_for_36_2

Genome browser session
(e.g. UCSC)

UCSC browser session: https://genome.ucsc.edu/s/tobiasfrie/HeLa_NSMB

Methodology

Replicates

2

Sequencing depth

Input_KO_42 60065014
Input_KO_44 67253460
Input_RBPJ_KO_42_Halo 62630434
Input_RBPJ_KO_42_HaloRBPJ_FL_AA 74134412
Input_RBPJ_KO_42_HaloRBPJ_K195E 61708938
Input_RBPJ_KO_42_HaloRBPJ_KRS 61084766
Input_RBPJ_KO_42_HaloRBPJ_R218H 62582028
Input_RBPJ_KO_42_HaloRBPJ_RFL_HAA 73028410
Input_RBPJ_KO_42_HaloRBPJ_WT 62111566

Input_RBPJ_KO_eV 62967412
Input_SHARP_KO_30 76775184
Input_SHARP_KO_30_Halo_only_1 71488336
Input_SHARP_KO_30_HaloRBPJ_WT_1 60729992
Input_SHARP_KO_36 62280630
Input_SHARP_KO_36_Halo_only_2 70142924
Input_SHARP_KO_36_HaloRBPJ_WT_2 66538146
Input_SHARP_KO_eV 71968460
RBPJ_KO_42_1 64381274
RBPJ_KO_42_2 80195412
RBPJ_KO_42_Halo_1 75077126
RBPJ_KO_42_Halo_2 61708008
RBPJ_KO_42_HaloRBPJ_FL_AA_1 68377666
RBPJ_KO_42_HaloRBPJ_FL_AA_2 62346276
RBPJ_KO_42_HaloRBPJ_K195E_1 77564188
RBPJ_KO_42_HaloRBPJ_K195E_2 63095978
RBPJ_KO_42_HaloRBPJ_KRS_1 72021354
RBPJ_KO_42_HaloRBPJ_KRS_2 65058142
RBPJ_KO_42_HaloRBPJ_R218H_1 77202802
RBPJ_KO_42_HaloRBPJ_R218H_2 61867588
RBPJ_KO_42_HaloRBPJ_RFL_HAA_1 70853804
RBPJ_KO_42_HaloRBPJ_RFL_HAA_2 62214854
RBPJ_KO_42_HaloRBPJ_WT_1 72076458
RBPJ_KO_42_HaloRBPJ_WT_2 63311180
RBPJ_KO_44_1 72014650
RBPJ_KO_44_2 67629362
RBPJ_KO_eV_1 73075570
RBPJ_KO_eV_2 74003070
SHARP_KO_30_1 91319682
SHARP_KO_30_2 81626444
SHARP_KO_30_Halo_only_1 64868926
SHARP_KO_30_Halo_only_2 70535484
SHARP_KO_30_HaloRBPJ_WT_1 64220704
SHARP_KO_30_HaloRBPJ_WT_2 77748260
SHARP_KO_36_1 62533460
SHARP_KO_36_2 64330422
SHARP_KO_36_Halo_only_1 63177654
SHARP_KO_36_Halo_only_2 71828926
SHARP_KO_36_HaloRBPJ_WT_1 62100382
SHARP_KO_36_HaloRBPJ_WT_2 65179148
SHARP_KO_eV_1 80183550
SHARP_KO_eV_2 84276336

Input_SHARP_KO_eV_for_36 71801706
SHARP_KO_eV_for_36_1 67839328
SHARP_KO_eV_for_36_2 68324116

Antibodies

RBPJ (Cell Signaling 5313)

Peak calling parameters

Peak calling was done using PeakRanger using default parameters and p- and q- values cutoffs of 0.0001 The resulting set was filtered against blacklisted chromatin regions, as detected by
ENCODE. Consensus peak sets were calculated by GenomicRanges' reduce function.

Data quality

Raw FASTQ files were inspected with fastqc. Trim_galore was used for quality aware trimming and removal of adapters using default parameters. Mapping and peak calling were evaluated by optical inspection in the genome browser as well as pair-wise correlation analysis of binding profiles (coverage).

Software

FastQC (https://www.bioinformatics.babraham.ac.uk/projects/fastqc/)
Trim Galore (https://www.bioinformatics.babraham.ac.uk/projects/trim_galore/)
hisat2 v.2.2.171
Integrative Genome Viewer (IGV) 2.8.9
PeakRanger 1.18
Picard Tools (https://broadinstitute.github.io/picard/)

R version 4.3.1 (2023-06-16)
Platform: x86_64-pc-linux-gnu (64-bit)
Running under: Ubuntu 20.04.6 LTS

```
Matrix products: default
BLAS:   /usr/lib/x86_64-linux-gnu/blas/libblas.so.3.9.0
LAPACK: /usr/lib/x86_64-linux-gnu/lapack/liblapack.so.3.9.0

locale:
 [1] LC_CTYPE=en_US.UTF-8       LC_NUMERIC=C               LC_TIME=de_DE.UTF-8        LC_COLLATE=en_US.UTF-8
LC_MONETARY=de_DE.UTF-8    LC_MESSAGES=en_US.UTF-8    LC_PAPER=de_DE.UTF-8
 [8] LC_NAME=C                  LC_ADDRESS=C               LC_TELEPHONE=C             LC_MEASUREMENT=de_DE.UTF-8 LC_IDENTIFICATION=C

time zone: Europe/Berlin
tzcode source: system (glibc)

attached base packages:
[1] grid      stats4    stats     graphics  grDevices utils     datasets  methods   base

other attached packages:
 [1] eulerr_7.0.0              ChIPpeakAnno_3.34.1       Gviz_1.44.0               dgof_1.4                  clusterProfiler_4.8.2
ChIPseeker_1.36.0
 [7] org.Mm.eg.db_3.17.0       GenomicFeatures_1.52.1    AnnotationDbi_1.62.2      gplots_3.1.3              Rsubread_2.14.2
edgeR_3.42.4
[13] limma_3.56.2              DESeq2_1.40.2             rtracklayer_1.60.0        systemPipeR_2.6.3         ShortRead_1.58.0
GenomicAlignments_1.36.0
[19] SummarizedExperiment_1.30.2 Biobase_2.60.0          MatrixGenerics_1.12.2     matrixStats_1.0.0         BiocParallel_1.34.2
Rsamtools_2.16.0
[25] Biostrings_2.68.1         XVector_0.40.0            GenomicRanges_1.52.0      GenomeInfoDb_1.36.1       IRanges_2.34.1
S4Vectors_0.38.1
[31] BiocGenerics_0.46.0       knitr_1.43

loaded via a namespace (and not attached):
  [1] splines_4.3.1                     BiocIO_1.10.0                     bitops_1.0-7                      ggplotify_0.1.1
  [5] filelock_1.0.2                    tibble_3.2.1                      polyclip_1.10-4                   graph_1.78.0
  [9] rpart_4.1.19                      XML_3.99-0.14                     lifecycle_1.0.3                   ensembldb_2.24.0
 [13] lattice_0.21-8                    MASS_7.3-60                       backports_1.4.1                   magrittr_2.0.3
 [17] rmarkdown_2.23                    Hmisc_5.1-0                       yaml_2.3.7                        plotrix_3.8-2
 [21] cowplot_1.1.1                     DBI_1.1.3                         RColorBrewer_1.1-3                zlibbioc_1.46.0
 [25] purrr_1.0.1                       AnnotationFilter_1.24.0           biovizBase_1.48.0                 ggraph_2.1.0
 [29] RCurl_1.98-1.12                   nnet_7.3-19                       yulab.utils_0.0.6                 VariantAnnotation_1.46.0
 [33] tweenr_2.0.2                      rappdirs_0.3.3                    GenomeInfoDbData_1.2.10           enrichplot_1.20.0
 [37] ggrepel_0.9.3                     tidytree_0.4.4                    codetools_0.2-19                  DelayedArray_0.26.7
 [41] DOSE_3.26.1                       xml2_1.3.5                        ggforce_0.4.1                     tidyselect_1.2.0
 [45] futile.logger_1.4.3               aplot_0.1.10                      farver_2.1.1                      viridis_0.6.4
 [49] base64enc_0.1-3                   BiocFileCache_2.8.0               jsonlite_1.8.7                    multtest_2.56.0
 [53] Formula_1.2-5                     tidygraph_1.2.3                   survival_3.5-5                    tools_4.3.1
 [57] progress_1.2.2                    treeio_1.24.2                     TxDb.Hsapiens.UCSC.hg19.knownGene_3.2.2 Rcpp_1.0.11
 [61] glue_1.6.2                        gridExtra_2.3                     xfun_0.39                         qvalue_2.32.0
 [65] dplyr_1.1.2                       withr_2.5.0                       formatR_1.14                      fastmap_1.1.1
 [69] latticeExtra_0.6-30               boot_1.3-28.1                     fansi_1.0.4                       caTools_1.18.2
 [73] digest_0.6.33                     R6_2.5.1                          gridGraphics_0.5-1                colorspace_2.1-0
 [77] GO.db_3.17.0                      gtools_3.9.4                      dichromat_2.0-0.1                 jpeg_0.1-10
 [81] biomaRt_2.56.1                    RSQLite_2.3.1                     utf8_1.2.3                        tidyr_1.3.0
 [85] generics_0.1.3                    data.table_1.14.8                 InteractionSet_1.28.1             prettyunits_1.1.1
 [89] graphlayouts_1.0.0                httr_1.4.6                        htmlwidgets_1.6.2                 S4Arrays_1.0.4
 [93] scatterpie_0.2.1                  regioneR_1.32.0                   pkgconfig_2.0.3                   gtable_0.3.3
 [97] blob_1.2.4                        hwriter_1.3.2.1                   shadowtext_0.1.2                  htmltools_0.5.5
[101] fgsea_1.26.0                      RBGL_1.76.0                       ProtGenerics_1.32.0               scales_1.2.1
[105] png_0.1-8                         ggfun_0.1.1                       lambda.r_1.2.4                    rstudioapi_0.15.0
[109] reshape2_1.4.4                    rjson_0.2.21                      checkmate_2.2.0                   nlme_3.1-162
[113] curl_5.0.1                        cachem_1.0.8                      stringr_1.5.0                     KernSmooth_2.23-22
[117] parallel_4.3.1                    HDO.db_0.99.1                     foreign_0.8-84                    restfulr_0.0.15
[121] pillar_1.9.0                      vctrs_0.6.3                       dbplyr_2.3.3                      cluster_2.1.4
[125] htmlTable_2.4.1                   evaluate_0.21                     VennDiagram_1.7.3                 futile.options_1.0.1
[129] cli_3.6.1                         locfit_1.5-9.8                    compiler_4.3.1                    rlang_1.1.1
[133] crayon_1.5.2                      interp_1.1-4                      plyr_1.8.8                        stringi_1.7.12
[137] viridisLite_0.4.2                 deldir_1.0-9                      munsell_0.5.0                     lazyeval_0.2.2
[141] GOSemSim_2.26.1                   Matrix_1.6-1.1                    BSgenome_1.68.0                   hms_1.1.3
[145] patchwork_1.1.2                   bit64_4.0.5                       ggplot2_3.4.2                     KEGGREST_1.40.0
[149] igraph_1.5.0.1                    memoise_2.0.1                     ggtree_3.8.0                      fastmatch_1.1-3
[153] bit_4.0.5                         downloader_0.4                    ape_5.7-1                         gson_0.1.0
```

Flow Cytometry

Plots

Confirm that:

- [x] The axis labels state the marker and fluorochrome used (e.g. CD4-FITC).
- [x] The axis scales are clearly visible. Include numbers along axes only for bottom left plot of group (a 'group' is an analysis of identical markers).
- [ ] All plots are contour plots with outliers or pseudocolor plots.
- [x] A numerical value for number of cells or percentage (with statistics) is provided.

Methodology

Sample preparation	Sample preparation is described in the method section "Flow cytometry to determine the cellular abundancy of HT-RBPJ-WT"
Instrument	Information about the instrument is given in the method section "Flow cytometry to determine the cellular abundancy of HT-RBPJ-WT". We used the Attune NxT Flow Cytometer blue/red/violet/yellow, Invitrogen no. A24858
Software	Attune(TM) Cytometric Software v5.3.0<br>FlowLogic version 8.7
Cell population abundance	At least 57 % of cells from the target cell line were included in the determination of mean fluorescence intensity.
Gating strategy	FSC-A/SSC-A gates were set in order to distinguish between live and dead cells.<br>FSC-H/FSC-A gates were set in order to exclude cell doublets.

- [ ] Tick this box to confirm that a figure exemplifying the gating strategy is provided in the Supplementary Information.
