# Supplementary material for: Effective in vivo binding energy landscape illustrates kinetic stability of RBPJ-DNA binding
Source: Nat Commun. 2025 Feb 1;16:1259. doi: 10.1038/s41467-025-56515-4 (PMC11787368; doi:10.1038/s41467-025-56515-4)
Supplement: Supplementary file 3 — Description of Additional Supplementary Files [file 41467_2025_56515_MOESM3_ESM.docx]

**Description of Additional Supplementary Files**

File Name: Supplementary Movie 1

Description: Example movie of HT-RBPJ-WT 3me-lapse imaging in a HeLa cell nucleus (dashed line) with 50 ms camera exposure 3me and 0.1 s frame cycle 3me. Related to Figure 2b. Scale bar: 4 μm.

File Name: Supplementary Movie 2

Description: Example movie of HT-RBPJ-WT imaging in a HeLa cell nucleus (dashed line) with con3nuous illumina3on at 11.7 ms frame cycle 3me. Scale bar: 4 μm
