# Supplementary material for: Effective in vivo binding energy landscape illustrates kinetic stability of RBPJ-DNA binding
Source: Nat Commun. 2025 Feb 1;16:1259. doi: 10.1038/s41467-025-56515-4 (PMC11787368; doi:10.1038/s41467-025-56515-4)

Western-Blot Figure S2a_upper:

IP: anti-Flag; WB: anti RBPJ








Western-Blot Figure S2a_lower:

IP: anti-Flag; WB: anti-Flag


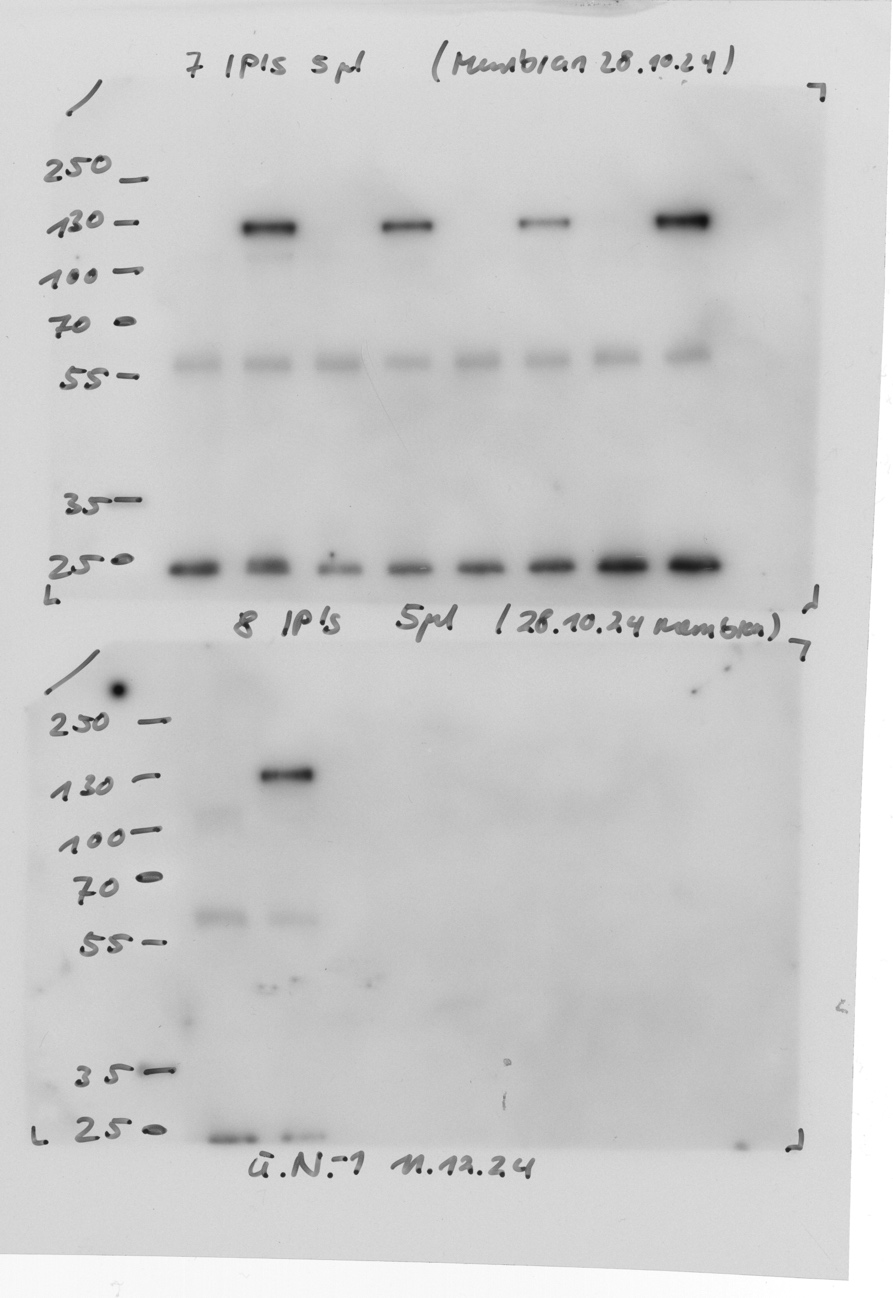





Western-Blot Figure S2b:

Upper: Input; WB: anti RBPJ





Western-Blot Figure S2b:

Lower: Input; WB: anti Flag





Western-Blot Figure S2c_upper:

IP: anti-Flag; WB: anti RBPJ








Western-Blot Figure S2c_lower:

IP: anti-Flag; WB: anti-Flag








Western-Blot Figure S2d:

Upper: Input; WB: anti-RBPJ





Western-Blot Figure S2d:

Lower: Input; WB: anti Flag





Western-Blot Figure S3b:

Upper: WB: Anti RBPJ


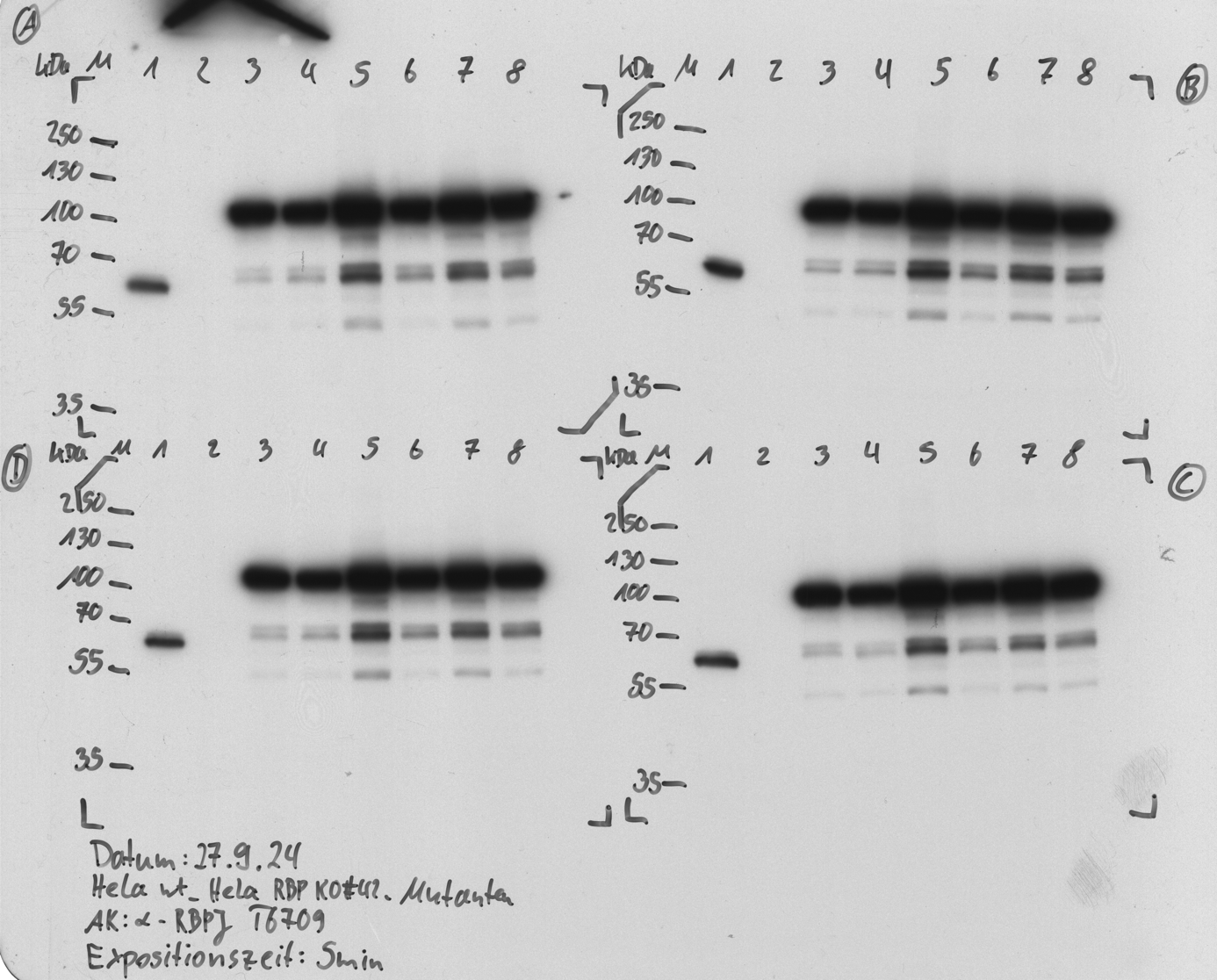


Western-Blot Figure S3b:

Lower WB_Loading Control: Anti beta-Actin


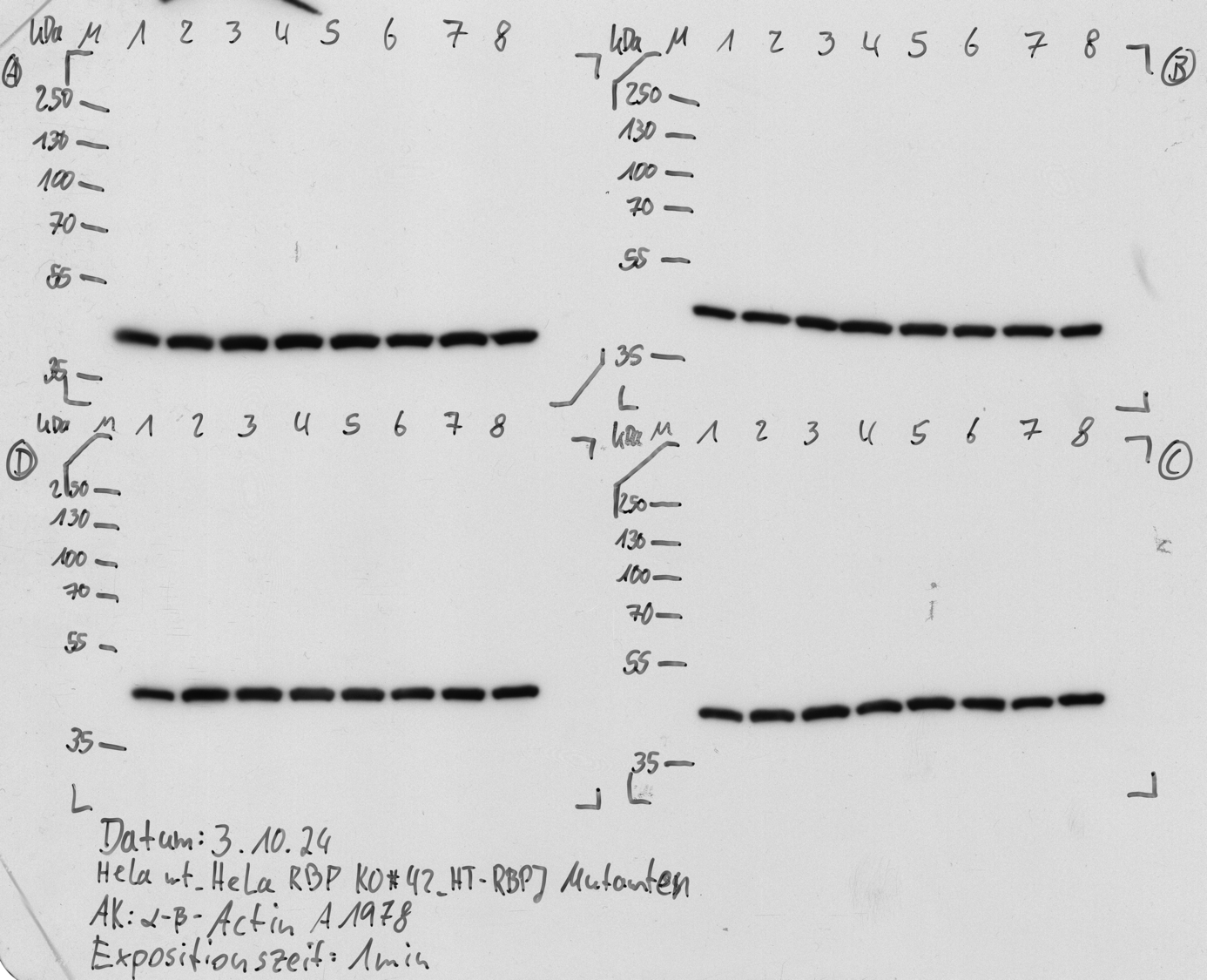


Western-Blot Figure S4b:

Upper panel:Long exposure_Anti-SHARP.1:


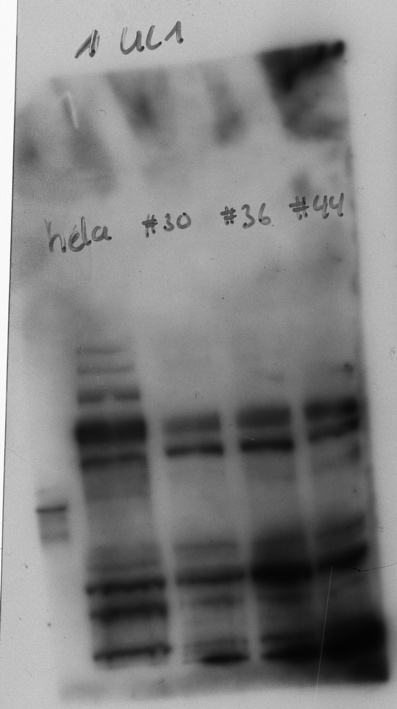


Lower panel: Short exposure_Anti-SHARP.1:


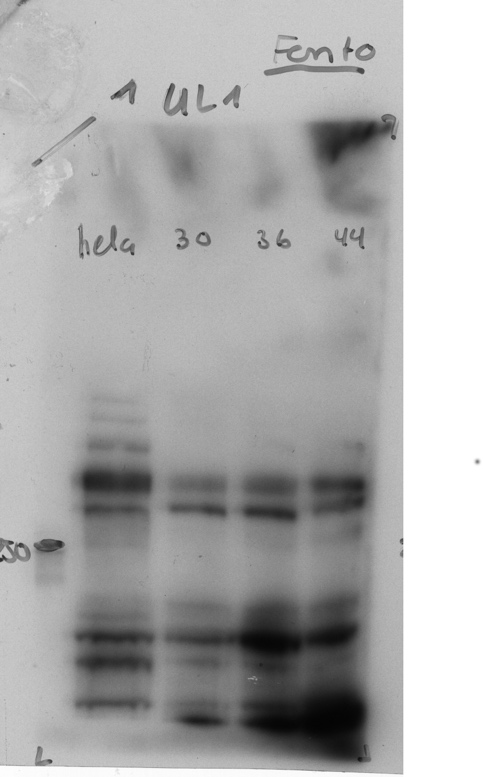


Western-Blot Figure S4g:

Upper panel_Anti-RBPJ:


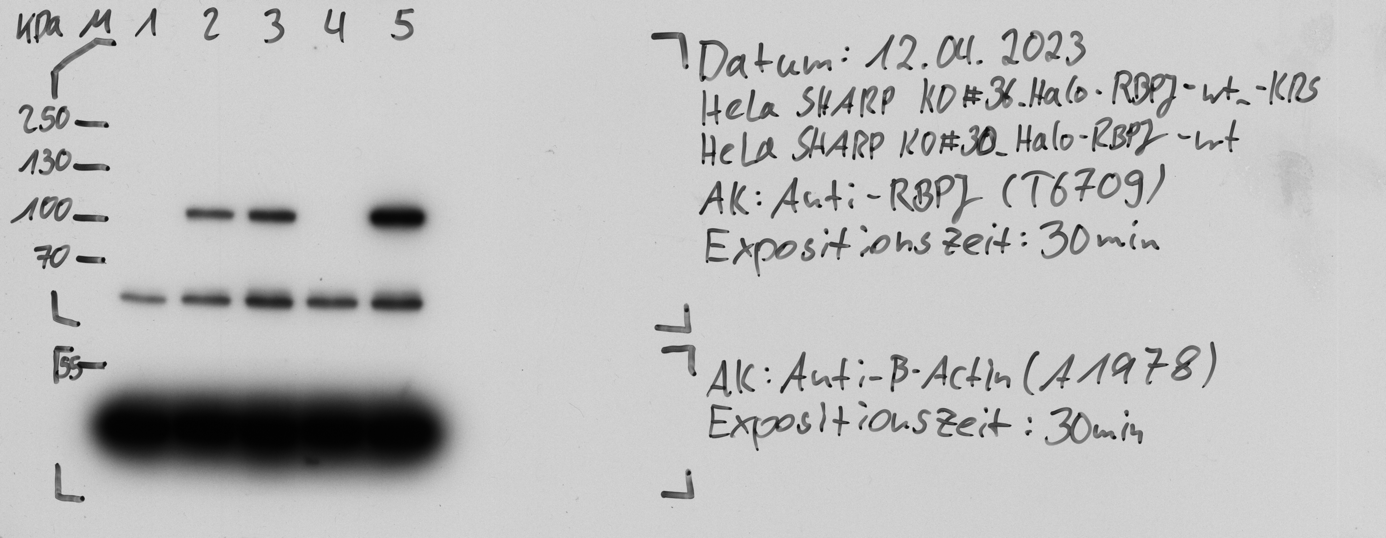


Lower panel_Loading Control Anti-Beta-Actin:


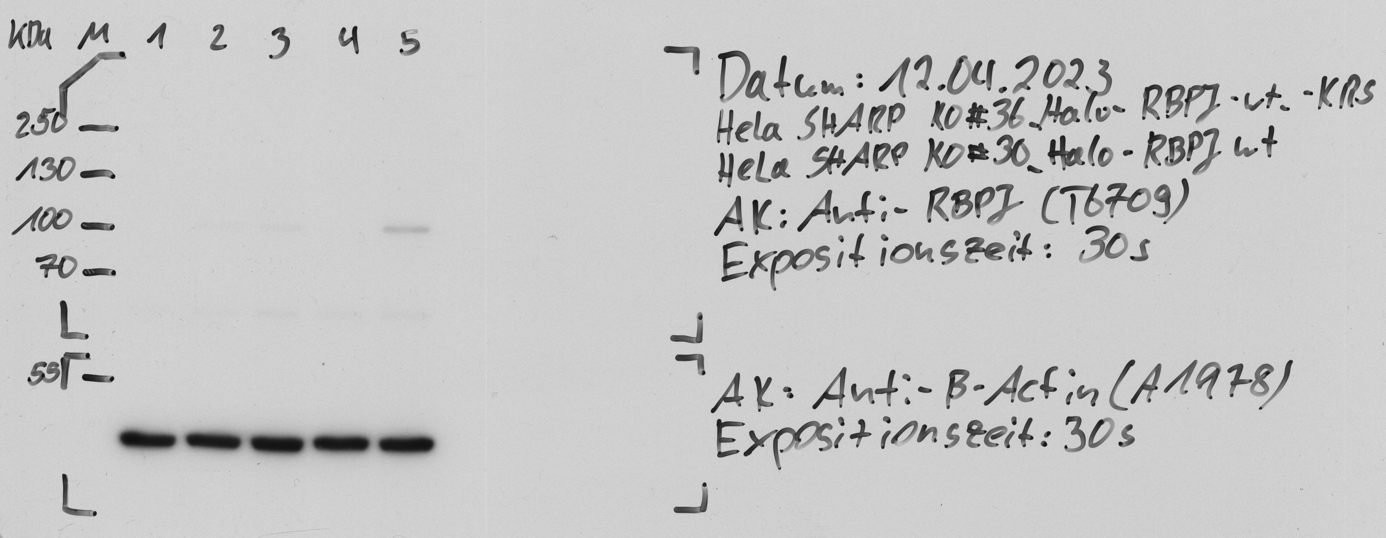


Western-Blot Figure S4h:

Upper panel: Anti-Halo-Tag


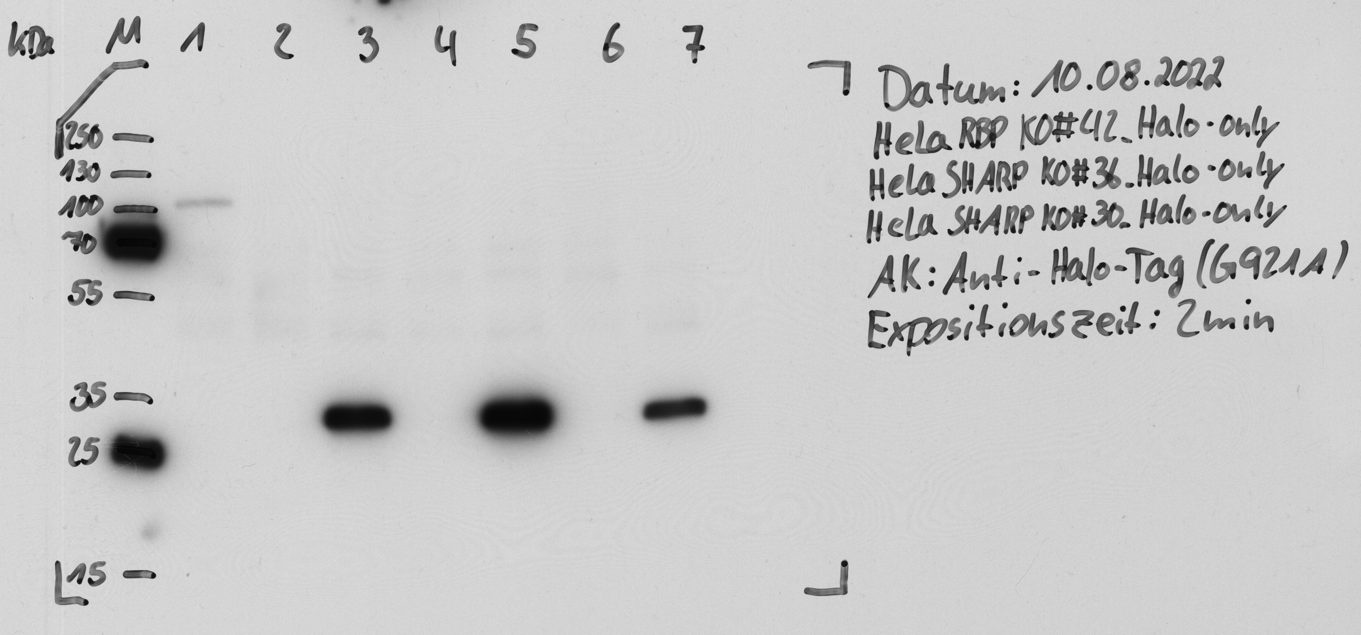


Lower panel_Loading Control_Beta-Actin:


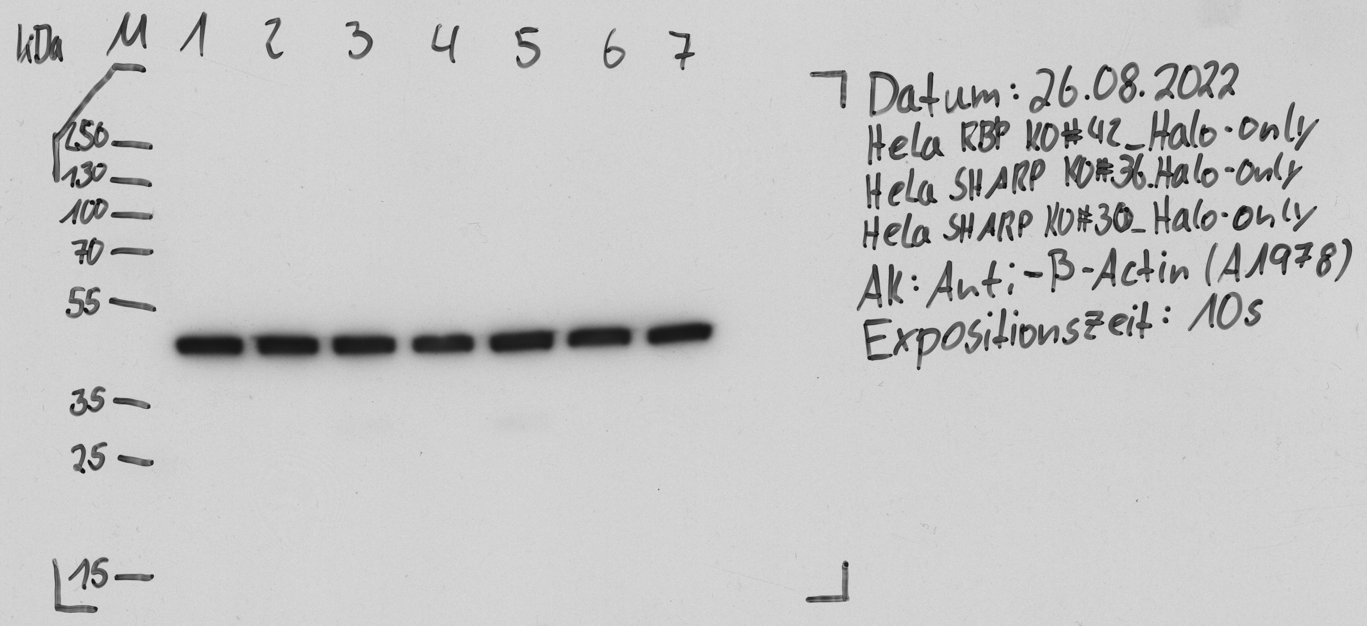


Supplementary Figure S8b:

Upper: WB: Anti RBPJ

Lower: WB_Loading control: Anti-beta-Actin


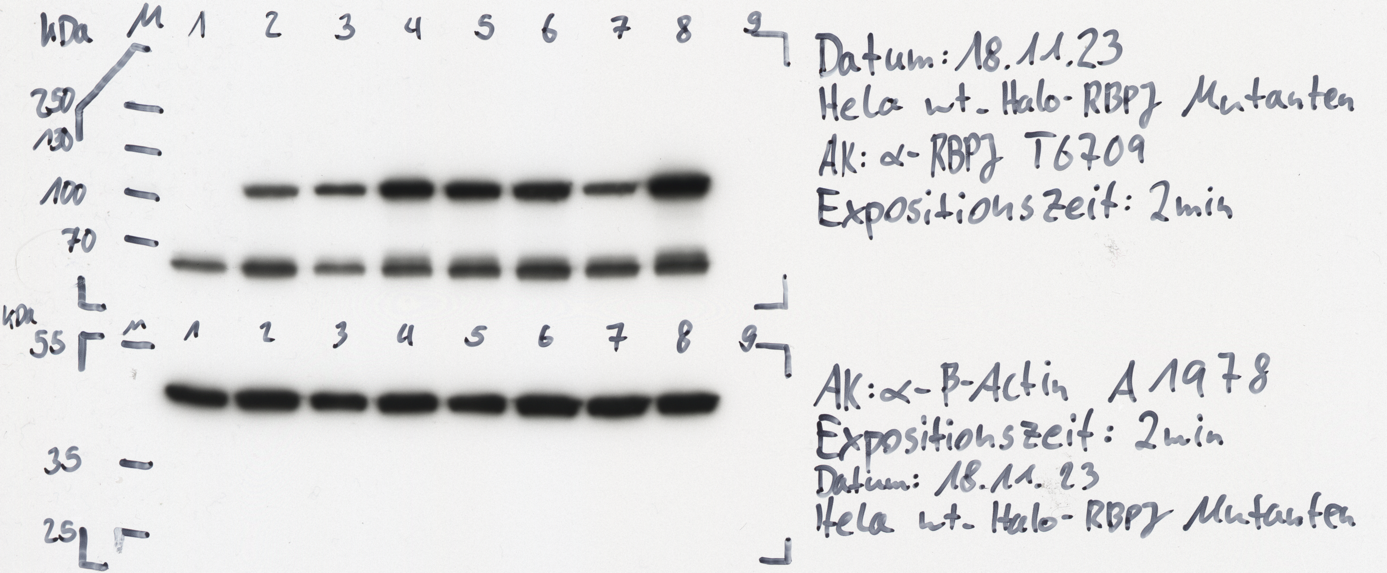


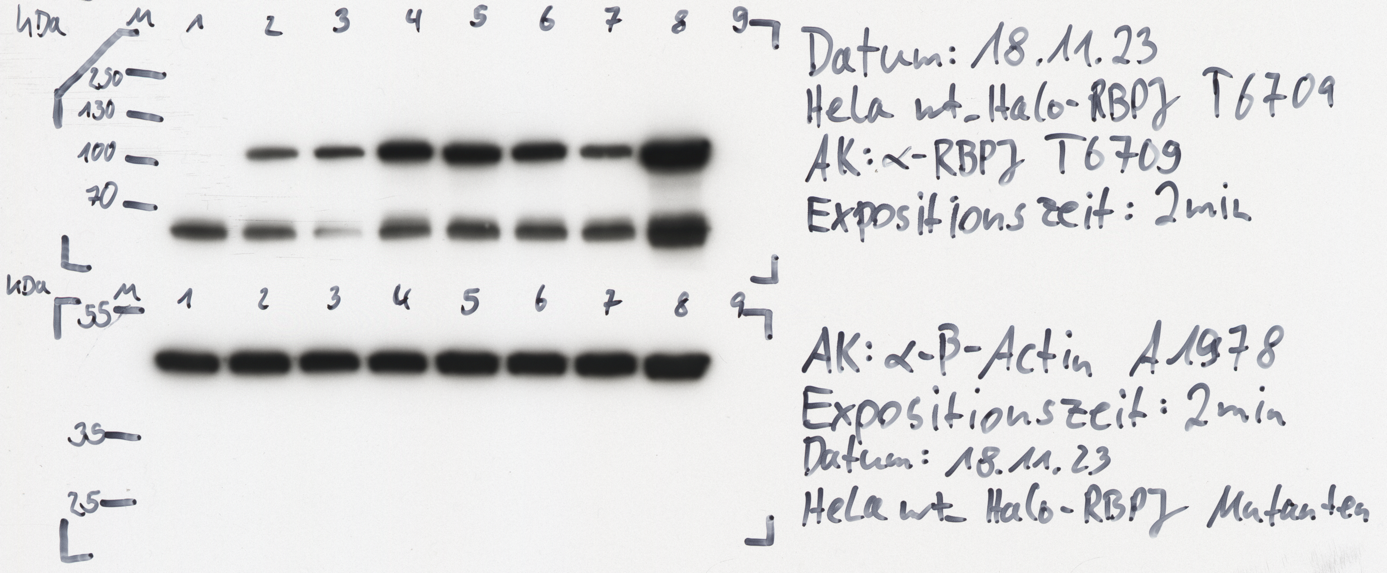

Supplement: Supplementary file 7 — Source Data [file 41467_2025_56515_MOESM7_ESM.zip › Source Data Western Blots.docx]
